# Projections of global-scale extreme sea levels and resulting episodic coastal flooding over the 21st Century

**DOI:** 10.1038/s41598-020-67736-6

**Published:** 2020-07-30

**Authors:** Ebru Kirezci, Ian R. Young, Roshanka Ranasinghe, Sanne Muis, Robert J. Nicholls, Daniel Lincke, Jochen Hinkel

**Affiliations:** 1 Department of Infrastructure Engineering, University of Melbourne, Melbourne, Australia; 2 Department of Water Science and Engineering, IHE-Delft, P.O. Box 3015, 2610 DA Delft, The Netherlands; 3 Harbour, Coastal and Offshore Engineering, Deltares, PO Box 177, 2600 MH Delft, The Netherlands; 4 Water Engineering and Management, Faculty of Engineering Technology, University of Twente, PO Box 217, 7500 AE Enschede, The Netherlands; 5 Institute for Environmental Studies (IVM), Vrije Universiteit Amsterdam, Amsterdam, The Netherlands; 6 Deltares, Delft, The Netherlands; 7 Tyndall Centre for Climate Change Research, University of East Anglia, Norwich, NR4 7TJ UK; 8 Global Climate Forum, 10829 Berlin, Germany; 9 Division of Resource Economics, Albrecht Daniel Thaer-Institute and Berlin Workshop in Institutional Analysis of Social-Ecological Systems (WINS), Humboldt-University, Berlin, Germany

**Keywords:** Physical oceanography, Climate-change impacts, Projection and prediction

## Abstract

Global models of tide, storm surge, and wave setup are used to obtain projections of episodic coastal flooding over the coming century. The models are extensively validated against tide gauge data and the impact of uncertainties and assumptions on projections estimated in detail. Global “hotspots” where there is projected to be a significant change in episodic flooding by the end of the century are identified and found to be mostly concentrated in north western Europe and Asia. Results show that for the case of, no coastal protection or adaptation, and a mean RCP8.5 scenario, there will be an increase of 48% of the world’s land area, 52% of the global population and 46% of global assets at risk of flooding by 2100. A total of 68% of the global coastal area flooded will be caused by tide and storm events with 32% due to projected regional sea level rise.

## Introduction

Sea level rise is a well-accepted consequence of climate change^1–3^. Although the focus of the general public often tends to be on the rate and magnitude of increase in mean sea level, the major threats of coastal flooding and erosion are significantly impacted by episodic storm surge and wave setup (the temporary increase in mean water level due to the presence of breaking waves) as well as their time of occurrence in relation to astronomical tide^4^. As approximately 600 million people live in low elevation coastal zones [i.e. LECZs—coastal regions less than 10 m above mean sea level (*MSL*)] which generate approximately US$1 trillion of global wealth^2,5–7^, both the environmental and socio-economic impacts associated with episodic coastal flooding can be massive.

Both national and global assessments of projected coastal flooding due to the combination of extreme events and sea level rise are critical in informing policy directions, as detailed in a number of IPCC reports. Such large-scale assessments can also identify regional “hot-spots” where more detailed modelling is required. The time and space scales involved in such large-scale assessment are challenging. The time scales vary from the duration of individual storms (of order hours) to projections over the coming century (of order decades). The physical scales are also demanding, varying from the bathymetry of individual beaches (10s of m) to basin-scale storms to global comparisons of potential impacts (1000s of km). As a result, to form tractable solutions and guide such policy development, a variety of simplifying assumptions must be made. This study aims to undertake such an analysis. It assembles extensive model and measured datasets at coastlines around the world and combines these to provide projections of global extreme sea level and coastal flooding by 2100. The required simplifications may result in local errors but comparisons with recorded tide gauge data indicates that, to first-order, the simplified model adopted reproduces extreme sea levels to reasonable accuracy at national and global-scale. The Discussion section below, and the Supplementary Material (SM5) provide a summary of the simplifications necessarily adopted to undertake this analysis and their potential implications.

In order to determine the frequency and magnitude of episodic coastal flooding, it is first necessary to determine sea levels during extreme storm events. The resulting extreme sea levels are generally made up of four components: tide (*T*), storm surge (*S*), wave setup (*WS*) and regional relative sea-level rise (*RSLR*). Projections of future coastal flooding require not only knowledge of the magnitude of each of these physical processes, but also their relative timing (i.e. does the storm occur at high tide) and an understanding of the probability of occurrence of extreme events. A number of recent studies have considered some of these elements, with and without validation against recorded data ^7–11^.

Consistent with previous global-scale studies^7–11^, it is assumed the total sea level (*TSL*) can be approximated by the linear summation:

1 $$ TSL(t) = T + S + WS. $$

where *TSL* is a function of time, $t$. The historical *TSL* estimates are rigorously validated against extensive global tide gauge data both for the historical record and for statistical extreme values. The term extreme sea level ($ESL_{T + S + WS}^{H100}$) is used here to represent the value of the *TSL* which occurs, for example, once in 100 years (a return period of 100 years). The superscript “*H*” signifies the extreme value is determined from the historical record, as opposed to a future projection and the subscript (*T* + *S* + *WS*) designates the physical processes considered in the determination of the extreme*.* For brevity, if the subscript is excluded, it signifies the use of all three processes ($ESL^{H100} \equiv ESL_{T + S + WS}^{H100}$). To obtain future projections, the extreme value estimates of *TSL* are coupled with global projections of *RSLR* for IPCC Representative Concentration Pathways (RCPs) 4.5 and 8.5. The resulting projected future extreme sea levels ($ESL_{{}}^{F100}$) are then used in conjunction with global topographic data to assess the potential extent of episodic coastal flooding at global scale in 2050 and 2100. As the projected flooding is sensitive to both how well the model dataset represents the physical processes and the appropriateness of the extreme value probability modelling, both of these elements are validated in detail (see SM2, SM3).

The global distribution of episodic coastal flooding is then used to determine global coastal flooding “hotspot” regions, where a significant increase in flooding is projected over the coming century. The relative contributions of each of the individual processes to coastal flooding are also assessed (i.e. surge, tide, wave setup and relative sea level rise), as are the projected changes in probability of occurrence of episodic flooding events in the future. Finally, we estimate the total population and value of exposed assets at risk both at present and in the future (2050 and 2100).

As noted above, as our focus is on an assessment at the global-scale, a number of local processes must be represented using approximations to render the problem tractable. We consider the limitations of these approximations and, where possible, estimate the sensitivity of the results to the inherent assumptions (see SM5). Statistical uncertainties associated with the projections are also estimated (see SM5).

This paper builds on previous studies of global-scale sea level rise. Importantly, the present study considers all three major processes, *T*, *S* and *WS*. It quantifies the relative importance of each of *S*, *WS* and *RSLR* processes to episodic coastal flooding by 2100. The modelling approach is extensively validated against tide gauge data for both ambient and extreme conditions. Extreme value estimates require statistical extrapolation of the data which can result in large confidence limits on estimates. The extreme value estimates are considered in detail, comparisons with recorded data undertaken and confidence limits estimated. Finally, the paper extends the sea level rise estimates to determine episodic coastal flooding extent, the populations impacted and the assets potentially at risk. Within the assumptions required to make such a global-scale study possible, it provides a “first-order” analysis forming a basis for future policy development.

## Results

### Datasets and processing

A detailed description of the datasets used in this study is provided in the “Methods” section. As the focus here is at the global-scale, *TSL(t)* over the period (1979–2014) was determined along global coastlines at a total of 9,866 points which approximate the coastal segments previously defined in the Dynamic Interactive Vulnerability Assessment database (DIVA)^12^ (see Fig. S1), referred to here as “DIVA points”. Historical values of surge ($S$) were determined over this period from the Global Tide and Surge Reanalysis (GTSR) dataset^8^. The tide levels (*T*) were determined from the numerical tide model dataset FES2014 (Finite Element Solution)^13^. In order to determine the wave setup, (*WS*), the nearshore (deep-water) wave conditions (significant wave height, $H_{s0}$ and wavelength, $L_{0}$) are required. As there is no widely validated and accepted global nearshore wave model dataset, two different reanalysis wave model datasets were tested for this purpose: ERA-Interim^14^ and GOW2^15^, with the latter ultimately being adopted (see SM1, SM2, Table S3). Wave setup was determined as a function of deep-water wave steepness ($H_{s0} /L_{0}$) and bed slope using the Shore Protection Manual (SPM) approach^16,17^. An alternative wave setup formulation proposed by Stockdon et al.^18^ was also tested and found to yield similar results (see SM1, SM2 and SM5). After testing a series of representative bed slopes, a value of 1/30 was finally adopted (see SM1, SM2). As each of the model datasets are on different global grids and at different temporal resolution, every DIVA point was assigned the value of the closest grid point for each model and the respective quantities of *T, S* and *WS* were interpolated in time to a 10-min resolution. The above approach does not include any contribution of wave run-up, consistent with the majority of published studies^7,9,11^, as run-up does not result in a sustained (order of hours) elevation of the *TSL.* This is in contrast to the recent study of Melet et al.^19,20^.

The historical time series of *TSL* over the period (1979–2014) was calculated using Eq. 1. This approach ignores non-linear interactions among these processes^8^. For instance, both surge and wave setup will be influenced by the phase of the tide. Comparison with measured tide gauge data suggests such interactions, at least at this global scale, do not appear to have a significant impact on the results (see SM1, SM2). Validation data over the historical period were obtained from the GESLA-2^21^ tide gauge dataset, which comprises sea level data at 681 locations around the world (see Fig. S1).

In order to determine coastal flooding extent, coastal topography data were obtained from the Multi-Error-Removed Improved-Terrain DEM (MERIT DEM) dataset^22^. Although the native resolution of the MERIT DEM is ~ 90 m at the Equator, a coarser 1 km resolution version, consistent with previous studies^8,23,24^ was used for the present application to reduce computational expense and ensure a resolution comparable to the other datasets used. MERIT is based on the SRTM v4.1 DEM dataset^25^, but with enhanced vertical accuracy (see “Methods” section).

In order to determine assets exposed due to flooding both gridded population and Gross Domestic Product (GDP) databases are required. Population data were obtained from the GPWv4 Rev. 11^26^ database and GDP data from Kummu et al.^27^.

### Historical global total sea level

Validation over the hindcast period is imperative for confidence in future projections. The model *TSL* time series was compared with the GESLA-2 tide gauge data over the period 1979–2014. Model performance over the hindcast period was evaluated at each of 681 GESLA-2 locations by determining both the root mean square error (*RMSE*) and the upper percentile bias ($bias^{p}$)*,* difference of higher percentile values (95th to 99th) between the model *TSL* and the tide gauge data. Overall global model performance was then assessed in terms of the average *RMSE* (*ARMSE*) and average $bias^{p}$ ($abias^{p}$) over all GESLA-2 locations^8^. The GESLA-2 tide gauge data were compared with both model *T* + *S* + *WS* and *T* + *S*. In addition, both GOW2 and ERA-Interim wave models, a variety of bed slopes and two different empirical formulations^16–18^ were used to calculate *WS*. The complete results are given in Tables S1 and S1 and discussed in SM1. As the differences between the various values of *ARMSE* and $abias^{p}$ are not large for the different combinations and because, as subsequently shown, *WS* is a relatively small component of the total episodic flooding, we confine our discussion here to cases where *WS* is calculated with the GOW2 model, the SPM^16,17^ formulation and the mid-range bed slope of 1/30. As noted above, the global-scale of the analysis meant that a relatively simplistic approach was necessarily used to determine *WS*^16,17^. As the results ultimately showed that *WS* was not a significant component of episodic flooding (5%, see SM3), errors caused by this approach are unlikely to significantly bias the final results.

For *T* + *S*, the globally averaged *ARMSE* is 0.197 m, which is comparable to the value of 0.170 m obtained by Muis et al.^8^, where an older tide model (FES 2012) was used together with a significantly smaller set of tide gauge locations (472). Inclusion of *WS* makes no appreciable change to *ARMSE,* in fact increasing it slightly to 0.204 m (see Table S1). This lack of impact on *WS* is not surprising, as *WS* is expected only to represent an appreciable contribution during storm events, which is poorly captured by *ARMSE*. The global distribution of values of *RMSE* for *T* + *S* + *WS* is shown at each GESLA-2 location in Fig. S2. Although there is an occasional outlier in the data, *RMSE* is less than 0.2 m at 75% of locations and less than 0.5 m at the vast majority (93%) of locations. The contribution of *WS* during storm periods (Figs. S8, S9) can be assessed from values of $abias^{p}$*.* Table S2, shows that for *T* + *S*, $abias^{p}$ increases in magnitude with increasing percentile level. With the addition of *WS*, $abias^{p}$ decreases, becoming approximately constant across all percentiles. The reduction in $abias^{p}$ is 60% at the 99th percentile, indicating the inclusion of *WS* results in better agreement between model and tide gauges during storm events. The improvement in $\left| {bias^{P} } \right|$, at individual tide gauge locations is shown in Fig. S4.

The validation outlined above indicates that the model derived *TSL* estimates are generally in good agreement with tide gauge data and that the inclusion of *WS* makes an improvement in performance, particularly during extreme storm events. As noted in SM1, it is unclear how many of the validation tide gauges respond to *WS* due to their locations. What is clear, however, is that without the inclusion of *WS*, there is a global underprediction of *TSL* during storms. Also, as shown in Fig. S4, the improvement in $\left| {bias^{P} } \right|$ can be seen at the vast majority of tide gauge locations. Whether this is actually due to *WS* or a systematic under prediction of *S* is not known. What is clear is that the inclusion of *WS*, modelled using the relatively simple approach adopted, results in a model which performs well compared to tide gauges at most locations.

### Extreme value estimates of total sea level

As noted above, both *S* and *WS* are episodic. For episodic coastal flooding, it is these storm-related contributions to extreme sea levels that are often critical^7,8,28,29^. The stochastic prediction of such extremes involves the fitting of an appropriate probability distribution function (pdf) to an historical time series and then extrapolating to the desired probability of occurrence (e.g. 0.01 in any year or the 100-year event). In the case of *TSL*, the most common approach has been to consider Annual Maxima (AM) and to fit either a two-parameter Gumbel distribution (GUM)^8,30^ or a three parameter Generalized Extreme Value distribution (GEVD)^7,30,31^. A significant limitation of AM approaches is that the resulting extreme value time series have few values (1 per year). This leads to relatively large confidence intervals when fitting and extrapolating the pdf. An alternative is to use all storm peaks above a specified threshold—i.e. the Peaks over Threshold approach, PoT^31,32^. In this latter case, the data can be shown to follow a Generalized Pareto Distribution (GPD)^32^ or its two-parameter variant, the Exponential Distribution (EXP). An alternative to the approach used above of reconstructing the long-term historical time series, is to use an ensemble Monte-Carlo approach^9^. This is discussed in SM4.

The Extreme Value Analysis (EVA) adopted can have a major impact on the resulting statistical estimates of extremes (in this case, extreme sea levels)^31^ (see Fig. S10). Therefore, it is important to ensure that the chosen EVA optimally approximates both the model and tide gauge data. Hence, a range of EVA approaches were tested to determine which optimally represents both model and tide gauge data (see SM2). Results indicate that the PoT approach fitted with a GPD and a 98^th^ percentile threshold (GPD98) fits both the tide gauge and model data with least error. This combination yields the best fit to the tide gauge data in 33% of locations and the best fit to the model data (at DIVA points) in 34% of locations (see Fig. S5). This result is consistent with the findings of Wahl et al.^31^. The complete EVA analysis is described in SM2.

A further analysis of the impact of the selected EVA approach on projected extreme sea level, as well as the sensitivity of the method used to determine *WS* is shown in Table S3. This table considers the mean bias between tide gauge and model results for a 20-year Return Period ($ESL^{H20} - ESL_{Gauge}^{H20}$) across the 355 (of a total of 681) tide gauge locations which have a duration of at least 20 years within the storm surge model time span (1979–2014). These results indicate a mean bias of 17 mm with the inclusion of *WS* determined from the GOW2 model, a 1/30 bed slope and a GPD98 EVA (see Fig. S7). However, a number of other combinations of EVA and *WS* calculation yield similar results. All the cases which include *WS*, have relatively small mean bias, indicating that the results are robust, irrespective of the choice of wave model, bed slope and EVA. What is clear, however, is that if *WS* is not included, there is a consistent negative bias (model underestimates the extreme sea level). For GPD98 with a 1/30 bed slope, the mean absolute bias is reduced by 88% indicating a significant improvement. Therefore, the inclusion of wave setup appears to produce model extreme sea levels ($ESL^{H20}$) that are in better agreement with recorded data.

**Figure 1:**
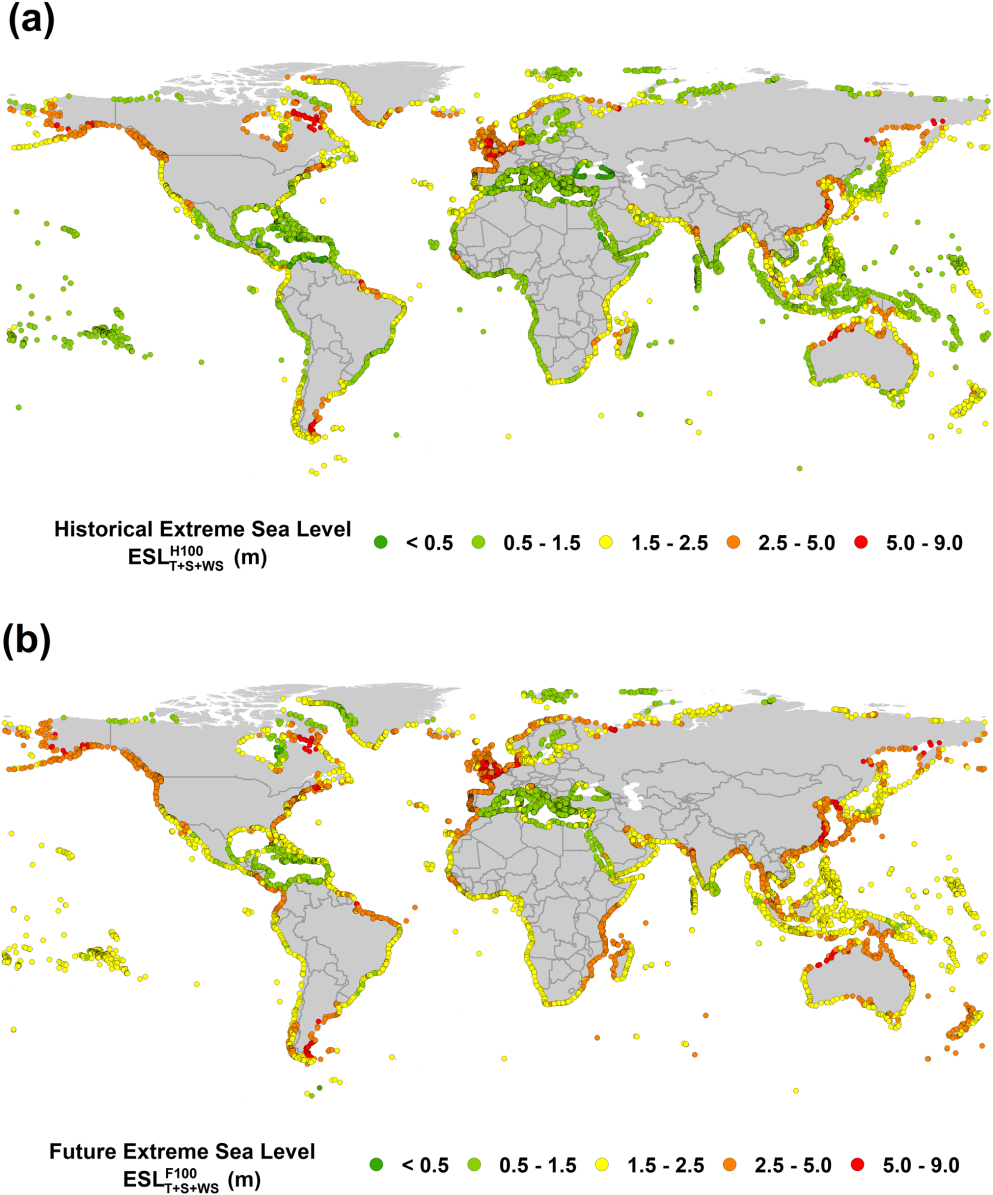
(**a**) Global distribution of the historical 100-year return period extreme sea level ($ESL_{T + S + WS}^{H100}$) at DIVA locations based on model data for the period 1979–2014. (Figure generated using ArcGIS v.10.5.1.7333, www.esri.com). (**b**)**.** Global distribution of projected 100-year return period extreme sea level ($ESL_{T + S + WS}^{F100}$) at DIVA locations for RCP8.5 in 2100 (figure generated using ArcGIS v.10.5.1.7333, www.esri.com).

With this validation of modelled $ESL^{H20}$, the results were extended to a return period of 1 in 100 years ($ESL^{H100}$) and evaluated at all DIVA points. The global distribution of $ESL^{H100}$ is shown in Fig. 1a. This figure shows that values in excess of 5 m occur along northern parts of both the Atlantic and Pacific coasts of North America, the Atlantic and North Sea coasts of Europe and China. The results show regional consistency with $ESL^{H100}$ varying gradually along coastlines. Note that these $ESL^{H100}$ estimates underestimate values in tropical cyclone regions due to model resolution^8^ and the limited sample size^33,34^.

Fig. S6 also shows the impact of *WS* alone, calculated as $ESL_{T + S + WS}^{H100} - ESL_{T + S}^{H100}$. This figure shows extreme *WS* values up to 0.5 m, with the distribution largely following areas of large extreme significant wave height^35^. In particular, the northern parts of both the Atlantic and Pacific coasts of North America, Atlantic coast of Europe, southern tip of the Pacific coast of South America, southern coast of Australia and much of Asia show 100-year return period contributions of *WS* greater than 0.4 m. Hence, although *WS* has only a very small impact on the overall values of *ARMSE TSL* between model and tide gauge data, it becomes a larger component where extreme value sea levels are concerned (on average a 17% increase in $ESL^{H100}$ due to *WS* over all DIVA points).

### Future projections of extreme sea levels and coastal flooding

The $ESL^{H100}$ values provide the basis to determine episodic flooding for the present day and for the future. The values of $ESL^{H100}$ at each DIVA point were associated with a surrounding region (see SM3) and flooding calculated using the following planar bathtub approach^8^. The topography was defined by the MERIT DEM dataset, which has a vertical datum of the EGM96 geoid (Earth Gravitational Model 1996). To bring values of $ESL^{H100}$ to this same datum, Mean Dynamic Ocean Topography (*MDOT*)^25,36^ values were added to the extreme value estimates ($ESL^{H100} + MDOT$)^23^*.* The coastline was defined using the Global Self-consistent Hierarchical High-resolution Geography (GSHHG) database^37^. A GIS-based approach was subsequently used whereby any MERIT grid point is considered inundated if it has an elevation less than $ESL^{H100}$ and is connected to the shoreline by water.

To calculate future Extreme Sea Level ($ESL^{F100}$), projected regional relative sea level rise (*RSLR*) was added to the present-day extreme sea level, $ESL^{F100} = ESL^{H100} + RSLR$*.* Values of *RSLR* vary by region around the world and were taken from Church et al. (Fig. 13.20—https://icdc.cen.uni-hamburg.de/1/daten/ocean/ar5-slr.html) ^1^for IPCC Representative Concentration Pathways (RCP) 4.5 and 8.5 (Note: average global *RSLR* across all the DIVA points is 0.21–0.71 m for RCP4.5 and 0.34–0.99 m for RCP8.5 by 2100). A range of other projected values of *RSLR* have been proposed, however, due to the overwhelming precedence of the IPCC projections, they have been adopted here. The values of *RSLR* include the effects of atmospheric loading, land ice, glacial isostatic adjustment (GIA) and terrestrial water sources. Figure 1b shows global values of $ESL^{F100}$ for 2100 under RCP8.5 (also see Fig. S12a for 2050 values). A comparison of Fig. 1a,b shows that by 2100, *T* + *S* + *WS* will still be a significantly larger contribution to extreme sea levels than relative sea level rise.

**Figure 2:**
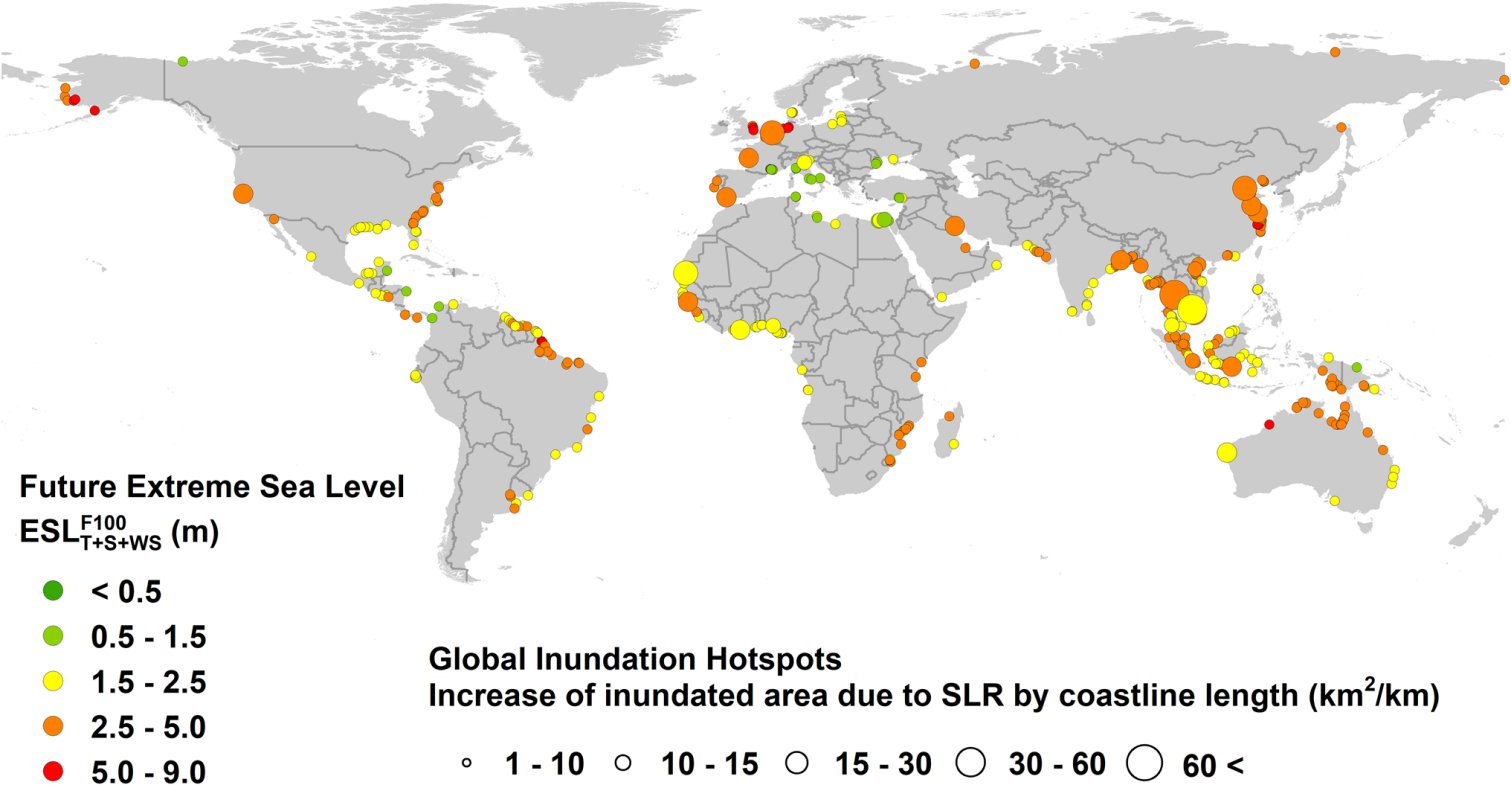
Global “hotspot” regions of changes in episodic coastal flooding in 2100 for RCP8.5. That is, the difference between projected episodic flooding in 2100 minus present day episodic flooding. Filled circles show locations where the change in normalized inundation (i.e. change in inundated area divided by length of coast) is greater than 1 km^2^/km. Size of circle is related to the change in magnitude of the normalized inundation. Colour of the circle is related to the projected extreme sea level in 2100 ($ESL_{T + S + WS}^{F100}$) (figure generated using ArcGIS v.10.5.1.7333, www.esri.com). *Note*: to add clarity, where points overlap, not every point is shown on the figure*.*

The extent of coastal flooding is a function both of $ESL^{F100}$ and the coastal topography. Figure 2 shows a global map of flooding “hotspot” regions in 2100 for RCP8.5. To arrive at this result, the flooding area per unit length of coastline was determined for each of the DIVA points (normalized inundation km^2^/km). The present analysis assumes there are no coastal defences (dykes, sea walls etc.). Therefore, rather than showing absolute values of inundation in 2100, Fig. 2 shows the change in inundation from the present to 2100. Areas with significant increases in flooding are seen in north-west Europe, India/Bay of Bengal, south-east and east Asia.

**Figure 3:**
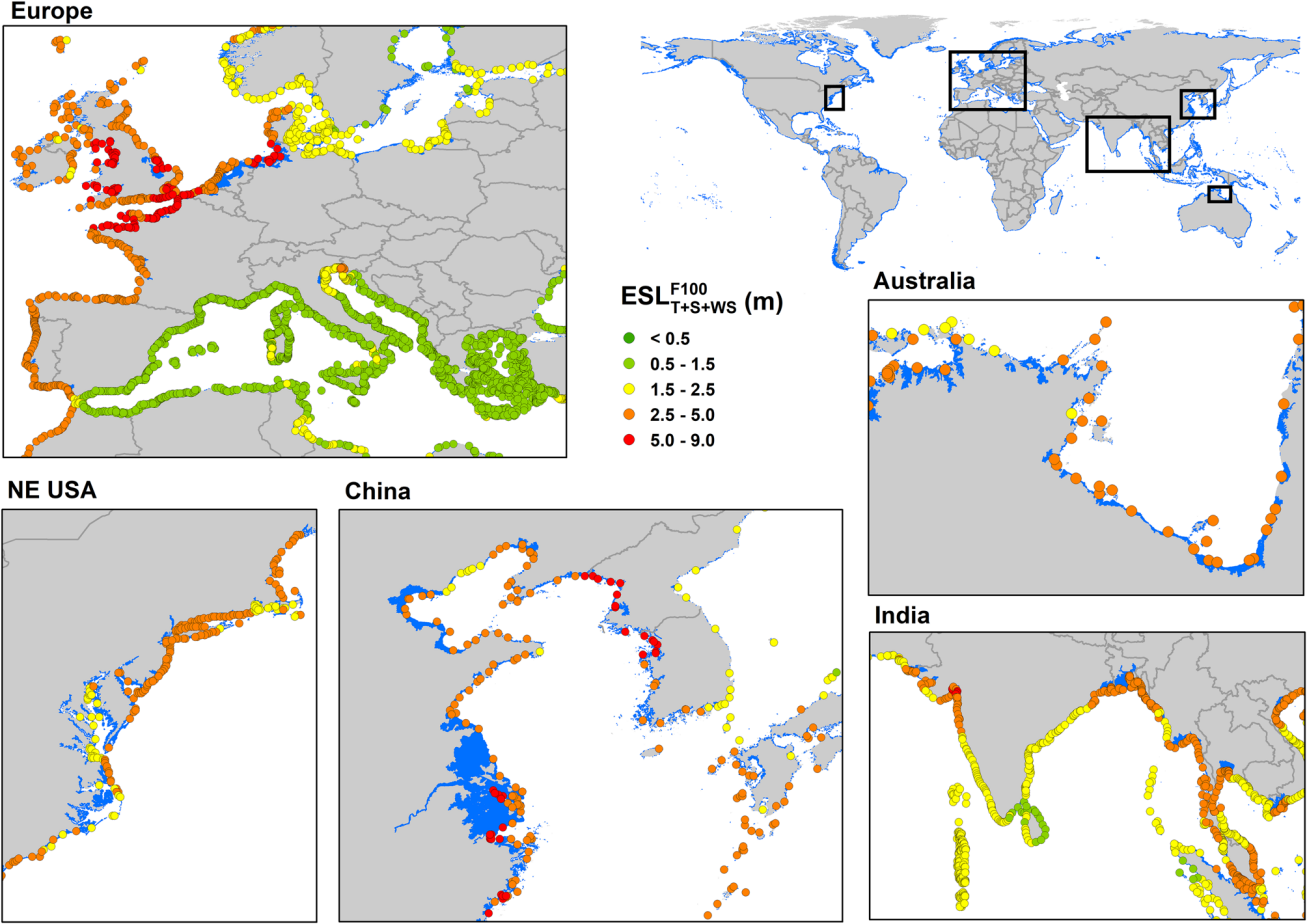
Regional areas showing the projected flooding associated with a 100-year return period extreme sea level event for 2100 (*T* + *S* + *WS* + *RSLR*). An RCP8.5 scenario is assumed. Coloured dots show the magnitude of the projected extreme sea level ($ESL_{T + S + Ws}^{F100}$) at the coast. Flooding extent shown by blue shading (figure generated using ArcGIS v.10.5.1.7333, www.esri.com).

Figure 3 shows both the $ESL^{F100}$ and the resulting flooding area for a number of the “hotspot” regions shown in Fig. 2. Although the flooding extent does not appear large in such plots, the global flooding extent for RCP8.5 is 661,000–1,009,000 km^2^ (approx. 0.5–0.7% of the global land area, larger than the land area of France). Note that the range of values represents the 90th percentile confidence interval (see “Methods” section). Table 1 shows the global flooding extent for each RCP for both 2050 and 2100. The Auxiliary Supplementary Data Google Earth file allows examination of values of $ESL^{H100}$ and $ESL^{F100}$ at any output location.

**Table 1:** Values of area of global episodic coastal flooding (with and without the wave setup contribution), population and assets exposed for different RCPs in 2050 and 2100. Present day values shown for comparison purposes. For each case the mean and lower and upper 90th percentile values are shown. Values in brackets represent the percentage change of mean values [ie. (future − present)/present].

		Inundated area (mean) without *WS* (10^3^ km^2^)	Inundated area with *WS* CL^0.5^–mean–CL^0.95^ (10^3^ km^2^)	Population exposed CL^0.5^–mean–CL^0.95^ (million people)	Asset exposed CL^0.5^–mean–CL^0.95^ (10^9^ US$2011)
**Present Day**		521	512–553–603	128–148–171	6,466—7,761–9,135
**2050**	RCP4.5	593 (14%)	549–631–721 (14%)	140–171–204 (16%)	7,094—8,848–10,672 (14%)
	RCP8.5	601 (15%)	560–640–732 (16%)	142–173–207 (17%)	7,188—8,961–10,799 (16%)
**2100**	RCP4.5	702 (35%)	604–737–894 (33%)	158–202–253 (36%)	7,919—10,222–12,739 (32%)
	RCP8.5	779 (50%)	661–819–1,009 (48%)	176–225–287 (52%)	8,813—11,301–14,178 (46%)

Further analysis of the relative contributions of the different physical processes to projected episodic coastal flooding (shown in Table 1) by the end of the twenty-first century (see SM3) indicates the following contributions for RCP8.5: *T* + *S* (63%), *RSLR* (32%), *WS* (5%). This result demonstrates that over the next century, *T* + *S* will remain the dominant process in determining the extent of global flooding. However, *RSLR* does significantly increase the frequency of coastal flooding. For RCP8.5, flooding associated with present day 100-year return period events will, on average, occur at least once every 10 years south of the 50°N latitude. It should be noted (see SM2) that the exact change in frequency of these extreme flooding events is sensitive to the EVA analysis used.

### Population and asset exposure

The global estimates of flooding described above, provide the basis for estimating both the population and the assets at risk from episodic coastal flooding. Asset exposure was estimated using the relationship^5,24^ $$A = 2.8 \times P \times G$$, where $$A$$ is the asset value exposed to flooding (US$), $$P$$ is the population and $$G$$ is the Gross Domestic Product per head of population (US$/head). As noted above, the population was estimated from the GPWv4 database^26^ and the GDP per capita from Kummu et al.^27^. Table 1 shows the area inundated together with the population and assets exposed for the present day, 2050 and 2100 under both RCP4.5 and 8.5. All values are in 2011 US$ and assume 2015 population and GDP, consistent with the databases used. To make a direct comparison between present day and future periods, no attempt to project changes in GDP or population in future years has been included here. The results project that the population potentially exposed to episodic coastal flooding will increase from 128–171 million to 176–287 million in 2100 under RCP8.5, where the span represents the 90th percentile confidence interval (see “Methods” section) (an increase from approximately 1.8–2.4% of the world’s population to 2.5–4.1%). The total assets exposed are projected to increase from US$6,466–US$9,135 billion to US$8,813–US$14,178 billion representing an increase from 9–13% to 12–20% of global GDP. As noted above, these values assume no flood defenses are in place and hence will overestimate the true values. However, the results indicate that for RCP8.5, by 2100 it is projected that the mean values of area inundated, population affected and assets threatened will increase by 48%, 52% and 46%, respectively.

## Discussion

Global model outputs of tide, surge and wave setup have been used to develop historical time series of total sea level around the world’s coasts. These model results were extensively validated against global tide gauge data, showing good agreement. To estimate the extreme sea levels which occur during storm events, an extreme value analysis was applied to both model and tide gauge data. In order to estimate extreme sea levels over the twenty-first century, projected relative sea level rise for IPCC RCP4.5 and 8.5 was added to present day extreme sea levels. These projected extreme sea levels were then used to quantify global episodic coastal flooding in 2050 and 2100.

Results show that for RCP8.5, 0.5–0.7% of the world’s land area will be at risk of episodic coastal flooding by 2100 from a 1 in 100-year return period event (an increase of 48% compared to the present day), impacting 2.5–4.1% of the world’s population (increase of 52%) and threatening assets worth up to 12–20% of global GDP (increase of 46%). Note that these values assume no coastal defences or adaptation measures (see SM5). In many locations, coastal defences are commonly deployed and by 2100, it is expected that adaptation and specifically hard protection will be widespread, hence these estimates need to be seen as illustrations of the scale of adaptation needed to offset risk. Future studies that consider the impact of coastal adaptation and defences could logically build on the present results. As such, we regard the present analysis as a “first-pass” estimate of global impacts of sea level rise.

The analysis shows that tide and storm surge will account for 63% of the global area inundated by 2100, with relative sea level rise accounting for 32% and wave setup accounting for only approximately 5%. Furthermore, projected sea level rise will significantly increase the frequency of coastal flooding by 2100, with results herein showing that for most of the world, flooding associated with a present day 1 in 100-year event could occur as frequently as once in 10 years, primarily as a result of sea level rise. As the episodic events of storm surge and wave setup will, between them, contribute approximately 68% of projected coastal flooding, any climate change driven variations in the frequency and/or severity of storm events could have significant impacts on future episodic coastal flooding.

As noted previously, the present study has a global-scale focus. As such, a number of simplifying assumptions are necessary to render the problem computationally feasible. These simplifications and the resulting implications are discussed in detail in the Supplementary Material (SM5). A summary of these assumptions appears below.

The analysis undertaken is linear in nature. It is assumed that the total sea level (*TSL*) can be represented by the summation of *T* + *S* + *WS.* This explicitly ignores interactions between these processes. Extreme value analysis is undertaken to determine historic extreme sea levels (*ESL*^*H*100^). The linear assumption is again applied to determine future extreme sea levels (*ESL*^*F*100^) by the linear addition of projected relative sea level rise (*RSLR*) by the end of 2100. This assumes that changes in wind speed and wave height over the coming century will be small, which is consistent with a number of recent studies^38,39,40^. SM5 outlines the precedence for such linear superposition approaches for global-scale studies^4,7,8,9,10,11^ and concludes that the potential errors are relatively small compared to the uncertainties in the extreme value analysis and *RSLR* projections.

In order to calculate the magnitude of the wave setup (*WS*) at global scale, it is necessary to use relatively simple models^16,18^ and to assume a global average bed slope. These assumptions will most likely result in errors at specific locations. However, the analysis ultimately shows that *WS* is a relatively small component of the *ESL*, and hence the adopted “first-order” representation of *WS* appears justified.

To validate the model adopted above, extensive tide gauge data is used (see Fig. S1), as this is the only global water surface elevation data source available. An extensive comparison is undertaken for both ambient and extreme conditions. It should be noted, however, that it is likely that many tide gauges, due to their locations, will not respond to *WS*. Hence, this validation dataset has its limitations for this application. Although model and tide gauge data agree well at the global scale, there are clear differences at specific locations. For example, in more than 30% of examined locations, the *RMSE* related to the mean tidal amplitude is greater than 20%. This is largely associated with semi-enclosed basins or regions with wide shelves (e.g. Mediterranean Sea, Baltic Sea, Sea of Japan) and with regions of small tidal range (see Fig. S3, SM1).

The MERIT^22^ topographic model is used with a “bathtub” flooding model. This assumption is expected to generally overestimate flood extent^41,42^. Importantly, the analysis also assumes no flood protection is in place, such as dykes or other structures. As a result, the absolute values of flood extent will be over-estimated in many locations. For this reason, we emphasis relative changes in flood extent rather than absolute values.

The above assumptions mean that the present analysis may not model projected flooding at specific sites well. However, results show that, when aggregated to the global scale, the approach adopted here is able to produce first-order estimates of global flooding and its implications. In addition to these simplifying assumptions, both the *ESL* and *RSLR* estimates have associated statistical uncertainties. The present study considered these uncertainties in assessing statistical variability associated with estimated flooding extent. The full analysis, given in SM5 and Table S4, indicates the uncertainty associated with projected flooding in 2100 (RCP8.5) is approximately $\pm 16.5\%$.

## Methods

**Figure 4:**
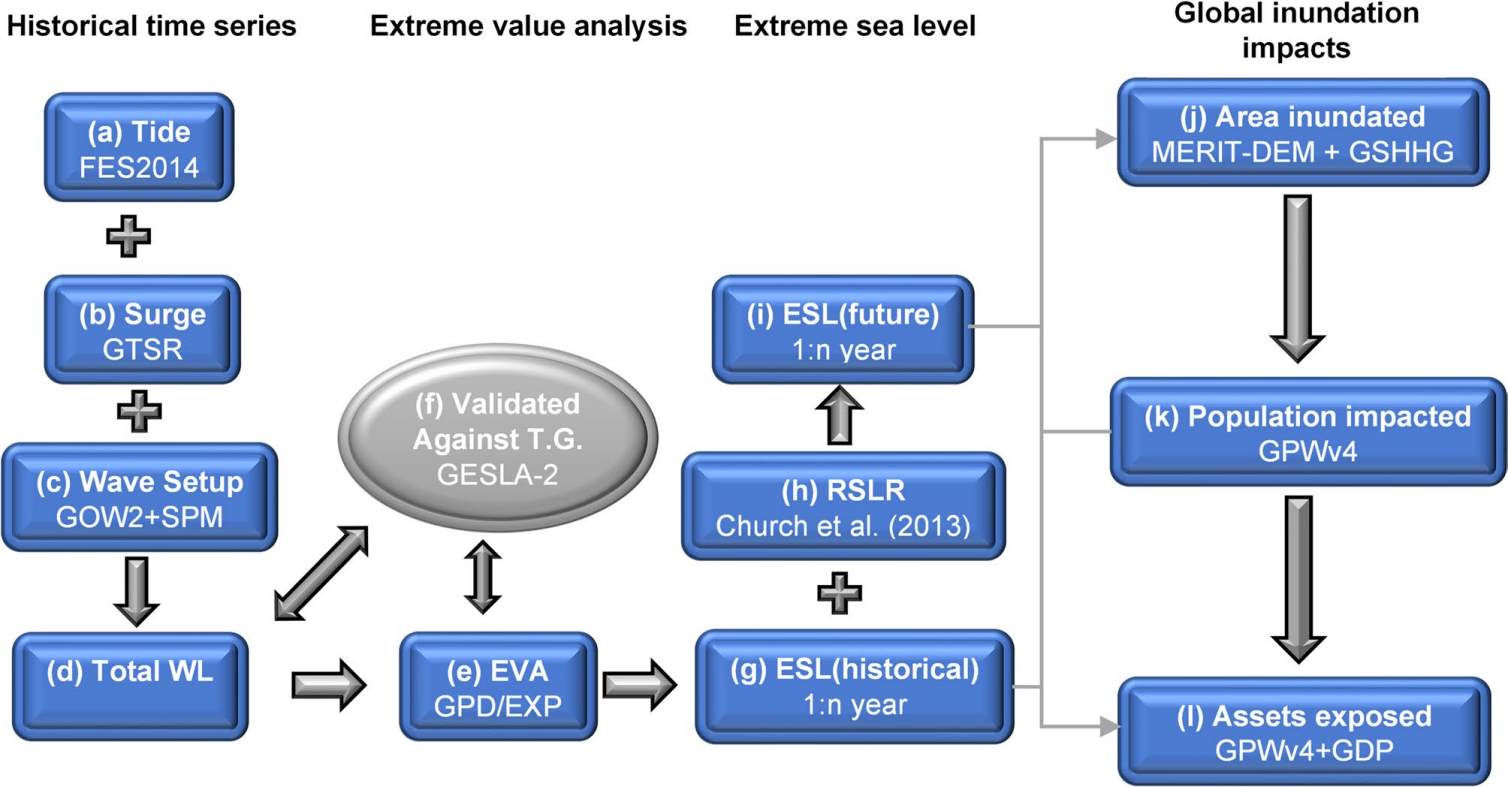
Diagrammatic representation of the processes used in the analysis of the various dataset in the full analysis. Terms and abbreviations defined in “Methods” section.

The analysis uses a significant number of global datasets which are combined to determine global projections of total sea level, extreme sea level, coastal flooding, populations affected and assets impacted for 2050 and 2100. The datasets are briefly described below and the process to combine and analysis them is shown diagrammatically in Fig. 4.

### Datasets

The various datasets used in this analysis are briefly described below:

### GTSR: Global tide and surge reanalysis (*S*)

The time series of coastal storm surge values were obtained from the GTSR dataset^8^ over the period (1979–2014). This dataset was generated with the Global Tide and Surge Model (GTSM), which uses the Delft3D Flexible Mesh software^43^ and was forced with wind fields from ERA-Interim^14^ downscaled to a temporal resolution of 10 min. GTSM has a spatially varying grid resolution which varies between 50 km in the deep ocean and 5 km in coastal areas.

### FES2014: Finite element solution (*T*)

FES is a finite element hydrodynamic model which solves the tidal barotropic equations and assimilates in-situ tide gauge and altimeter data^13^ (https://www.aviso.altimetry.fr/en/data/products/auxiliary-products/global-tide-fes/description-fes2014.html). The global model has a total of 2.9 M nodes and a spatial resolution of 1/16°.

### ERA-interim, GOW2 (*WS*)

ERA-Interim and GOW2 are global surface wave model reanalyses. These have been used to provide deep water wave conditions to estimate wave setup (*WS*). ERA-Interim (ERA-I) is a global atmospheric reanalysis from 1979^14^. ERA-I uses the ECMWF Cy31r2 atmospheric model coupled with the WAM spectral wave model^44^. Both the atmospheric model and the wave model used in ERA-I incorporate satellite data assimilation. Output from the model is available at 0.7° global resolution. GOW2^15^ is a global wave model hindcast. It uses the Wavewatch III^45^ model Version 4.18, forced with CFSR winds^46^. The hindcast is designed to provide higher resolution coastal wave data and hence uses a system of nested grids of resolution 0.5° in deep water, with finer scale ~ 25 km resolution in areas with water depths less than 200 m. In contrast to ERA-I which has satellite assimilation in both the wind and wave models, GOW2 has only assimilation in the forcing wind field. GOW2 hindcast data are available over the period 1979–2015.

In order to determine the wave setup, (*WS*), the nearshore (deep-water) wave conditions (significant wave height, $H_{s0}$ and wavelength, $L_{0}$) are required. As there is no widely validated and accepted global nearshore wave model dataset, both ERA-Interim^14^ and GOW2^15^ were tested for this purpose. Wave setup was determined as a function of deep-water wave steepness ($H_{s0} /L_{0}$) and bed slope using both the SPM graphical approach^16,17^ and Stockdon et al.^18^. Bed slope was calculated from an offshore depth equal to $2H_{s0}$ to the shoreline^17^. The spatial resolution of the wave models is such that bed slope cannot be determined accurately for the present application (i.e. ERA-Interim approx. 80 km resolution and GOW2 ~ 25 km resolution). However, as the dependence on bed slope is expected to be relatively weak^17^, a range of representative values (1/15, 1/30, 1/100) which spans reported global shoreline bed slopes^47,48^ were tested instead (see SM1 and SM2). Based on the outcomes of this sensitivity analysis (see SM2), a representative bed slope of 1/30 was adopted at all locations. Also, as GOW2 has better spatial and temporal resolution than ERA-Interim, and as wave setup estimates derived from the two wave models are similar (see SM1, SM2, Table S3), GOW2 was used for subsequent analysis.

### GESLA-2: Global extreme sea level analysis version 2 (*tide gauge data*)

GESLA-2^21^ is a dataset of global tide gauge observations. Although some of the tide gauges go back more than 100 years, the vast majority of the data are from 1950 onwards. Data are generally archived at a temporal resolution of 1 h or less and are available at a total of 1,355 stations. In the present analysis a total of 681 unique locations with data over the required period (see Fig. S1) were used to validate the various model results.

### DIVA: Dynamic interactive vulnerability assessment (*output locations*)

DIVA^12^ is a database for assessing coastal vulnerability from sub-national to global levels. The database, as such, is not used in this analysis. Rather, 9,866 DIVA locations have been used as the reference output locations for model results.

### MERIT DEM: Multi‐error‐removed improved‐terrain DEM (*Topography*)

MERIT DEM^22^ is a high accuracy global digital topographic dataset at 3 arc sec resolution (~ 90 m at equator) developed from existing spaceborne DEMs [SRTM3 v2.1 and AW3D-30 m v1^25,49^] by eliminating major error components from the existing DEMs. The DEM data covers lands between 90°N–60°S, vertically referenced to the EGM96 geoid.

### MDOT: Mean dynamic ocean topography (*MERIT DEM datum*)

The MDOT^36^ is the difference between the time-averaged sea surface and the geoid. As the datum for the estimates of $ESL^{H100}$ is mean sea level and datum for the MERIT DEM topography is the geoid, the MDOT was used to bring these datasets to the same datum.

### GSHHG: Global self-consistent hierarchical high-resolution geography (*Coastline*)

GSHHG^37^ is a coastline dataset at multiple resolutions. Here, the “high resolution” (~ 0.2 km) coastline dataset was used to define the global coastline for calculations of flooding extent.

### GPWv4 Rev.11: Global population *(population and asset exposure)*

The NASA Socioeconomic Data and Applications Center (SEDAC) produce the GPWv4 Rev. 11 database of population count from 2015 census data on a 30 arc sec. (~ 1 km at equator) grid. This dataset was used to determine population and assets potentially exposed to flooding.

### GDP: Gross domestic product *(asset exposure)*

The gridded 2015 GDP data of Kummu et al.^27^ consists of GDP per capita (PPP) data on a 5 arc min grid (downscaled to 30 arc-sec to be consistent with the other datasets used). This dataset was used to estimate the value of assets potentially exposed to flooding.

### Analysis process

Consistent with the linear assumption (1), the time series of *T* (Fig. 4a), *S* (Fig. 4b) and *WS* (Fig. 4c) obtained from the respective datasets were interpolated to a consistent 10-min temporal resolution and assigned to the closest DIVA point. This generated an historical time series of *TSL(t)* at each point. The *WS* was estimated using both the SPM approach^16^ and Stockdon et al.^18^, both GOW2 and ERA-I wave models were tested with a range of different bed slopes. The Stockdon et al.^18^ approach is more sensitive to bed slope but for plausible bed slopes (i.e. < 1/30) consistent with SPM^16^ (e.g. see Fig. S11). Based on subsequent global comparisons with tide gauge data, a bed slope of 1/30 was adopted with the SPM approach. The *RMSE* was used to test the consistency of these model-derived time series at each location against tide gauge data (Fig. 4f) for ambient conditions (see SM1). The performance of the model results for extreme conditions was also tested by comparing upper percentile values with the tide gauges (Fig. 4f).

The 1 in 100-year return period extreme value sea levels (*ESL*^*H*100^) were determined from these model time series at each DIVA point (Fig. 4e). A wide range of different extreme value analyses (EVA) were tested. These include the peaks-over-threshold method with both Generalized Pareto Distribution (GPD) and the Exponential Distribution (EXP) and a variety of different threshold levels. The Annual Maximum approach was also tested using the Generalized Extreme Value Distribution (GEV) and Gumbel distribution (GUM) (Fig. 4e). These approaches were validated against corresponding EVA analysis of tide gauge data. These comparisons were undertaken for 1 in 20-year return periods, which require no extrapolation of the time series to this probability level and 1 in 100-year return periods (see SM 2). Based on this analysis it was found that a GPD distribution with a 98^th^ percentile threshold gave the best agreement between model and tide gauges and inclusion of *WS* slightly reduced bias between model and tide gauge extreme value estimates (see SM2).

The projected future extreme sea level (*ESL*^*F*100^) (Fig. 4i) was determined by adding the relative sea level rise (*RSLR*) to *ESL*^*H*100^(Fig. 4h). Again, this was done at each DIVA point. Using a bathtub flooding assumption, the episodic coastal flooding was determined at each DIVA point with the *ESL* values assigned to areal regions using Thiessen polygons (see SM3). The coastal topography was determined using the MERIT digital elevation model with the coastlines defined using GSHHG dataset (Fig. 4j).

The population impacted by these flooded regions was determined from the gridded population data of the GPWv4 dataset (Fig. 4k) (see SM3). The value of assets impacted by the flooding was evaluated from the population impacted and the GDP using the relationships proposed by Hallegatte et al.^5^ and Hinkel et al.^24^ (Fig. 4l).

### Confidence limits

**Figure 5:**
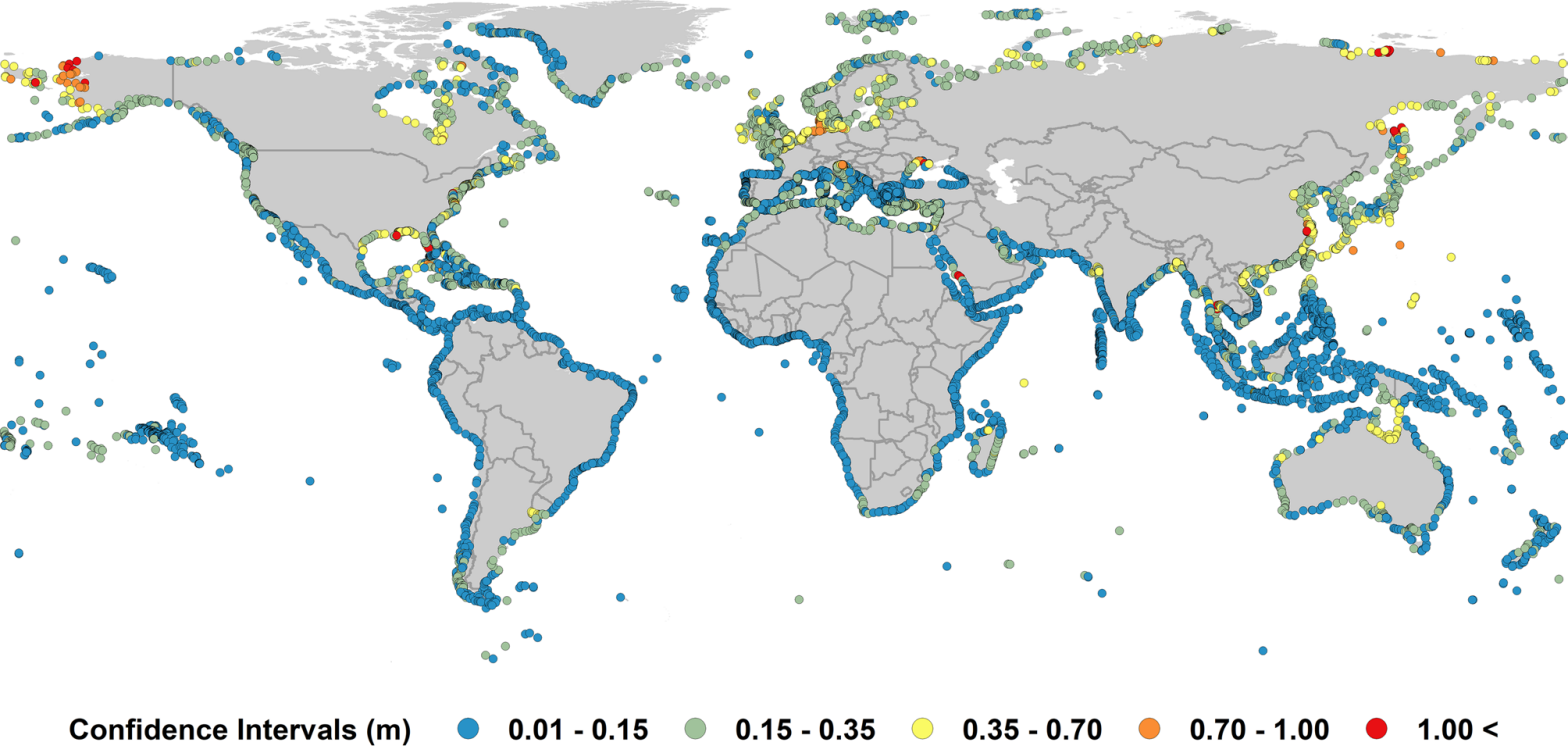
The 90th percentile confidence interval for present-day extreme sea level estimates ($ESL_{T + S + WS}^{H100}$) (i.e. upper confidence limit—lower confidence limit) (figure generated using ArcGIS v.10.5.1.7333, www.esri.com).

The estimates of extreme value sea levels are statistical quantities and to obtain estimates of the potential uncertainty in the projected $ESL^{F100}$, the 90^th^ percentile confidence limits on each of the $ESL^{H100}$ values were determined using a bootstrap approach. Bootstrapping is a common approach to determine confidence limits for extreme value estimates^40,50^. Using this approach, we computed a sample of 1,000 bootstrapped $ESL^{F100}$ estimates taken randomly from the original data sample at each DIVA point. For each sample an estimate of $ESL^{F100}$ was determined and 5.0 percentile and 95.0 percentile values calculated from the 1,000 realizations to give the lower and upper 90^th^ percentile confidence limits. The resulting 90th percentile confidence intervals are shown globally in Fig. 5. The results indicate that for 99% of the 9,866 locations, the span of the 90^th^ percentile confidence interval (i.e. upper *CL*—lower *CL*, *CL* is the value of $ESL^{H100}$ at the confidence limit) is less than 1 m (i.e. $\pm 0.5$ m). As $ESL^{F100}$ values are commonly of order 4 m (see Fig. S12), the 90^th^ percentile confidence limits are thus less than $\pm 10\%$ (see SM5). The confidence limits for $ESL^{H100}$ and $ESL^{F100}$ were used to determine the confidence limit span for area inundated, population exposed to flooding and assts exposed to damage. These values are shown in Table 1.

## Supplementary information

Supplementary file1 (DOCX 4564 kb)

Supplementary file2 (KMZ 479 kb)

## Data Availability

The data availability is outlined in “Methods” section.
