# Supplementary material for: Projections of global-scale extreme sea levels and resulting episodic coastal flooding over the 21st Century
Source: Sci Rep. 2020 Jul 30;10:11629. doi: 10.1038/s41598-020-67736-6 (PMC7393110; doi:10.1038/s41598-020-67736-6)
Supplement: Supplementary file 1 — Supplementary file1 (DOCX 4564 kb) [file 41598_2020_67736_MOESM1_ESM.docx]

Supplementary Materials for

**Projections of global-scale extreme sea levels and resulting episodic coastal flooding over the 21^st^ Century**

Ebru Kirezci^1^, Ian R. Young^1*^, Roshanka Ranasinghe^2,3,4^, Sanne Muis^5,6^, Robert J. Nicholls^7^, Daniel Lincke^8^, Jochen Hinkel^8,9^

1. *Department of Infrastructure Engineering, University of Melbourne, Melbourne, Australia.*
2. *Department of Water Science and Engineering, IHE-Delft P.O. Box 3015 2610 DA Delft, The Netherlands*
3. *Harbour. Coastal and Offshore Engineering, Deltares, PO Box 177, 2600 MH Delft, The Netherlands*
4. *Water Engineering and Management, Faculty of Engineering Technology, University of Twente, PO Box 217, 7500 AE Enschede, The Netherlands*
5. *Institute for Environmental Studies (IVM), Vrije Universiteit Amsterdam, Amsterdam, The Netherlands*
6. *Deltares, Delft, The Netherlands*
7. *Tyndall Centre for Climate Change Research, University of East Anglia, Norwich NR4 7TJ, UK.*
8. *Global Climate Forum, 10829 Berlin, Germany*
9. *Division of Resource Economics, Albrecht Daniel Thaer‐Institute and Berlin Workshop in Institutional Analysis of Social‐Ecological Systems (WINS), Humboldt‐University, Berlin, Germany*

*Correspondence to: [ian.young@unimelb.edu.au](mailto:ian.young@unimelb.edu.au)

**This PDF file includes:**

Text

References 51 - 68

Figs. S1 to S12

Table S1 and S4

Auxiliary Supplementary Data:

Google Earth file of Extreme Total Sea Level at DIVA sites

1. **Contributions of processes to Total Sea Level**

As noted in the main paper, model estimates of *T+S+WS* were validated against the GESLA-2 tide gauge data. This was achieved by determining both the *RMSE* and the $bias^{p}$(difference) of higher percentile values (95^th^ to 99^th^) between the model *TSL* and the tide gauge data at each of the 681 GESLA-2 tide gauge locations. GESLA-2 data points ( 21) used in this study are shown in Fig. S1. GESLA-2 provides a more extensive validation dataset than the University of Hawaii Sea Level Center (UHSLC) dataset used by Muis et al. ( 8). Both the *T* and *S* models use Mean Sea Level as their datum, when these quantities are summed, there can be a small mean offset due to the *S*. To obtain consistent datum levels, the annual means were subtracted from both the tide gauge and *T+S* model data at each of the GESLA-2 data points ( 8). As values of *WS* are always positive, adding *WS* to the model *T+S* dataset results in a positive offset. This was again removed by subtracting the annual mean from the *T+S+WS* model dataset. This validation implicitly assumes that the tide gauges respond to *WS*. Whether individual gauges do respond to wave setup will depend on their individual locations and the spatial extent of enhanced sea surface elevation resulting from the *WS*. Note that, when we subsequently use model values to determine extreme sea levels and flooding, the annual mean is not subtracted from the time series. Rather, in those cases a common absolute datum is obtained from the MDOT (see Future Projections of Extreme Sea Levels and Coastal Flooding section of the main paper).

As shown in Fig. S2, the *RMSE* at individual GESLA-2 data locations is generally less than 0.5m. Such absolute values of *RMSE* can mask the relative errors for locations where the tidal amplitude is small. In such cases, a small absolute *RMSE* may still be important. Fig. S3 shows the *RMSE* at each tide gauge location, presented as a percentage of the mean tidal amplitude. The mean tidal amplitude was determined by firstly performing a harmonic analysis on the tidal gauge data, so as to extract the astronomical tide. A zero up-crossing analysis was then performed to determine the mean amplitude of the tidal signal. This figure confirms what is shown in Fig. S2 and shows that at the majority of locations (68%) the relative *RMSE* is less than 20%. The locations where the relative *RMSE* is larger are enclosed basins (e.g. Mediterranean Sea, Baltic Sea, Sea of Japan) where the tidal amplitude is small and the spatial scales challenge our global-scale analysis, probably due to the performance of the tidal model.

Fig. S4 shows that there is generally an improvement in the 99^th^ percentile *bias^P^* (reduction in value) when *WS* is added to the model time series. Table S1 shows values of *ARMSE* (average *RMSE* over all tide gauges) and *abias^P^* (average bias of the 99^th^ percentile over all tide gauges). In this table, results are given for *T+S* (i.e. no *WS*) as well as *T+S+WS*, with *WS* calculated from both the ERA-I and GOW2 wave models and for a number of different bed slopes (1/15, 1/30 and 1/100). As *WS* is only significant during episodic storm events, *ARMSE* is hardly impacted by the inclusion of *WS*. The values of *ARMSE* change by only approximately 4% with the inclusion of *WS*. In fact, the agreement between model and tide gauge data is slightly worse with the inclusion of *WS*. The impact of *WS* is, however, larger for *abias^P^*, which examines the average 99^th^ percentile differences between model and tide gauges. The inclusion of *WS* reduces the magnitude of *abias^P^* by approximately 60%. This indicates that the inclusion of *WS* allows the model to more accurately reproduce the tide gauge data during storm conditions. As shown in Fig. S4, this reduction in bias is not limited to specific geographic locations. Rather, there is a consistent reduction in the magnitude of the bias at the 99^th^ percentile across 73% of tide gauge locations. The improvement in bias across all locations is further seen in Fig. S6, which shows histograms of *bias^P^* both with and without *WS* included. The inclusion of *WS* results in a more peaked histogram centred on a value of zero. When *WS* is excluded, the histogram is skewed to negative values, indicating an underestimation of extremes. As can be seen in Fig. S4, this improvement occurs in most global regions, not only tropical cyclone areas, where the GTSM is known to underestimate surge levels.

Table S2 shows values of *abias^P^* both with and without *WS* for a number of percentile levels (95^th^ to 99^th^ percentiles). For the case of *T+S* (no *WS*), the magnitude of *abias^P^* increases with percentile level. The inclusion of *WS* (*T+S+WS*) reduces the magnitude of *abias^P^* and also results in an almost constant value, as a function of percentile level. This indicates that the inclusion of *WS* in the model results in a better approximation of the low probability tail of the pdf.

*WS* is sensitive to local bed slope and bathymetry which cannot be resolved by the present wave models, not even with the finer resolution GOW2 model. As our focus is at a global scale, these effects can only be modelled in the mean. As seen in Table S1, there is very little difference in *ARMSE* or *abias^P^* between the ERAI and GOW2 models and for different bed slopes. Noting this, we have adopted the GOW2 model and a medium-range bed slope of 1/30 for subsequent calculations. The sensitivity of this choice for extreme value analysis is discussed in SM2.

The storm-related impact on *TSL* is highlighted in Figs. S8 and S9 for two specific locations (Boston, east coast USA) and Fremantle (south-west coast Australia). In each case the *T* component has been removed from the measured GESLA-2 sea level via a harmonic analysis. The resulting residual sea level is then compared with model *S* and *S+WS*. Each figure shows both residual sea levels and GOW2 significant wave height ($H_{s}$) over a 12-month period. A specific storm event for each location is also expanded for a more detailed analysis. These figures clearly show the episodic storm-related nature of *S* and *WS*. They also show that during these storm events, better agreement occurs between measured and model results when *WS* is included. Surge alone underestimates the residual sea level. In both of these cases, the tide gauges appear to respond to *WS*, which is well modelled by using the present approach ( 16, 17), the GOW2 model wave height and a bed slope of 1/30.

1. **Extreme Value Validation**

Although the ability of the modelling approach to reproduce long term *TSL* variations at tide gauge locations is desirable, it is the ability to reproduce extreme sea levels which is most important for the determination of episodic coastal flooding. To this end, a number of different extreme value approaches were investigated to determine which best modelled the tail of the probability distribution functions of both the model and tide gauge data. Ten different Extreme Value Analysis (EVA) approaches were tested. These include two Peaks over Threshold (PoT) fits to the data [Generalized Pareto Distribution (GPD) and Exponential (EXP)] and two Annual Maxima (AM) fits [Gumbel (GUM) and Generalized Extreme Value (GEV)]. Each of the PoT approaches was evaluated with 4 different threshold values, each determined as a percentile value (98^th^, 98.5^th^, 99^th^ and 99.5^th^) (i.e. 2x4 PoT approaches plus 2 AM approaches). These theoretical extreme value distributions were fitted to the GESLA-2 tide gauge data at a total of 355 stations for which there is at least 20 years of data. The goodness of fit of the chosen distribution was determined by examination of Q-Q relationships between tide gauge data and each theoretical distribution. The *RMSE* between tide gauge and theoretical distributions were evaluated for data above the 80^th^ percentile. A small *RMSE* indicates that the tail of the observed pdf is approximated well by the assumed distribution. The lower bound of 80% was adopted as it is high enough to ensure the tail of the pdf is being considered but not so high that comparisons become unacceptably noisy due to limited data. A range of other values (up to the 95^th^ percentile) were tested and produced the same conclusions. This approach allows a ranking of the various approaches over all tide gauge locations. Fig. S5 shows a global plot of tide gauge locations and which EVA approach best fits the data. A histogram of the number of locations where each EVA approach produces the best fit to the tide gauge data is also shown. This figure shows that a GPD98 PoT pdf best approximates the tide gauge data, consistent with the findings of Wahl et al ( 31). The same analysis was also undertaken for the model (*T+S+WS*) data (at DIVA points), yielding a similar result and confirming that the model data are also best approximated by the GPD98 approach.

It is clear in Fig. S5 that the GPD performs better than the EXP distribution. This is not surprising, as the GPD approaches are all 3-parameter pdfs, whereas the EXP distributions are 2-parameter pdfs. The additional parameter in the GPD providing greater flexibility in modelling the data. The same influence can be seen in the AM distributions, where the 3-parameter GEV pdf performs better than the 2-parameter GUM pdf.

The *RMSE* analysis of the Q-Q plots provides a quantitative method to rank how well the various EVA approaches approximate the tails of the pdfs. An alternative approach is to evaluate the bias between the tide gauge and model at a specified return period. As the tide gauge stations considered all have measurement durations greater than 20 years, a return period of 20 years was chosen to avoid errors due to extrapolation ($ESL^{H20}$). Table S3 shows the average bias between tide gauge and model ($ESL^{H20}-ESL_{Gauge}^{H20}$) across all 355 locations. The first point to note is that all EVA approaches yield a negative bias when the model *TSL* is approximated by *T+S* (i.e. no *WS*). This negative bias is largely eliminated when *WS* is included. With the inclusion of *WS*, all of the EVA approaches yield average bias values less than approximately 50mm. This result is irrespective of the EVA approach and wave model used to calculate the *WS* or assumed bed slope. As noted in the Q-Q *RMSE* analysis (Fig. S5), the GPD98 with GOW2 wave model and a bed slope of 1/30 performs well with a mean bias of approximately 17 mm. The impact of *WS* on the $ESL^{H20}$ values is clearly shown in Fig. S7, which shows a histogram of bias between model and tide gauge, both with and without *WS*. Note that this figure was evaluated with a GPD98 EVA, the GOW2 wave model and a bed slope of 1/30 to determine *WS*. Without the inclusion of *WS*, the $ESL^{H20}$model results underestimate the extreme values compared to the tide gauge data.

Although the combination of a GPD98 EVA, the GOW2 wave model and a bed slope of 1/30 to determine *WS* produced good agreement with the tide gauge derived predictions of extreme sea levels, a number of the other approaches yield similar or even better results in Table S3 (e.g. GPD98.5/GOW2-100). The fact that the average bias is relatively insensitive to the EVA approach and method for determining *WS* provides confidence in the robustness of the $ESL^{H20}$model values. However, the fact that there is not a single bed slope which clearly minimizes errors makes the selection of this value challenging. Athanasiou et al. ( 47) compiled data globally and found a mean value of approximately 1/100. Ardhuin and Roland ( 48), however, in a study of wave reflection from shorelines found much steeper slopes and adopted values as steep as 1/5. Based on this study, Melet et al. ( 19) adopted a global value of 1/10. Noting these discrepancies, we have pragmatically adopted a value of 1/30. This value lies between the estimates of Athanasiou et al. ( 47) and Ardhuin and Roland ( 48) and is consistent with the results in Table S3. The *WS* increases with increasing bed slope ( 16, 17, 18). Therefore, adopting a value of 1/30 over a value of 1/100 will produce larger $ESL^{100}$ values. The ultimate contribution of *WS* to total $ESL^{H100}$is approximately 17% on average but makes an even smaller contribution to global area flooded (approximately 5%). Therefore, we conclude that assuming a bed slope of 1/30 has negligible impact on the final results. Considering the results of both the *RMSE* and average bias tests and the discussion above, the GPD98/GOW2-30 combination was selected as the reference for subsequent analysis.

Although the results indicate that all EVA approaches provide comparable results at the 20-year return period, as shown by Wahl et al ( 31), they can yield quite different values when extrapolated to larger return periods. This is demonstrated in Fig. S10 for the location of Boston. This figure shows the $ESL^{H}$ calculated using GPD98/GOW2-30 and EXP98/GOW2-30 as a function of return period. Both model and tide gauge $ESL^{H}$are shown for a number of return periods and for the model cases, results both with and without the inclusion of *WS* are considered. As expected from the above analysis, the GPD results (model – tide gauge) are in better agreement than with the EXP distribution. Also, model $ESL^{H}$is underestimated when *WS* is not included. The Boston location is qualitatively representative of other sites.

As shown by Wahl et al ( 31) most of the locations (85%) considered have a negative shape parameter for the GPD99. This means that the distribution is bounded and, as shown in Fig. S10 is quite flat as a function of return period. Therefore, as return period increases, there is only a relatively small increase in $ESL^{H}$. In contrast, the 2-parameter EXP distribution is unbounded and has a much faster increase in $ESL^{H}$as a function of return period. For the GPD case, this means that a small change in $ESL^{H}$results in significant changes in equivalent return period.

Note that for the GPD98 EVA, there was typically between 800 and 1000 points (extremes) in the fit to the tail of the pdf.

1. **Determination of flooding and contributions of various processes**

Values of $ESL^{H100}$and $ESL^{F100}$have been estimated at each of the DIVA output points. In order to determine resulting episodic coastal flooding, these values need to be associated with a surrounding area. The association of areal significance to point measurements occurs in a number of disciplines, most notably hydrology. Following these precedents, we assigned Thiessen polygons to each DIVA point. The boundaries of such polygons are defined by lines whose perpendicular bisectors are equidistant between surrounding points. With the regions associated with each DIVA point determined, and the topography within the polygon defined by the MERIT elevations, locations which were shore-connected and where $ESL^{H100}$or $ESL^{F100}$ were greater than the land elevation were considered to be inundated.

The flooding extent in Table 1 provide the basis to determine the relative contributions to episodic coastal flooding of each of the physical process of *T*, *S*, *WS* and *RSLR*. Table 1 indicates that for the present day, the total area inundated is 553x10^3^km^2^, which reduces to 521 x10^3^km^2^ if *WS* is excluded. Hence, *T*+*S* contributes 94.2% (521/553) and *WS* the remaining 5.8%. For 2100 and RCP8.5, the total area inundated increases to 819 x10^3^km^2^ (*T*+*S*+*WS*+*RSLR*) and if *WS* is excluded this area decreases to 779 x10^3^km^2^. Therefore, *WS* contributes 4.9% [(819-779)/819)], *RSLR* 32.5% [(819-553)/819] and *T*+*S* the remaining 62.6%. Therefore, the present analysis shows that by the end of the 21st century, the largest contribution to extreme sea level and flooding will be the combined impacts of *T+S*. This will be responsible for approximately 63% of flooding, *RSLR* for RCP8.5, 32% and *WS* 5%. As noted in the main text, these coastal flooding calculations assume no coastal defences such as dykes or sea walls.

1. **Comparison with previous studies**

In this study, we have gone to considerable lengths to extensively validate at global-scale the approach against a comprehensive tide gauge dataset ( 21) for both ambient and extreme conditions, so as to be able to obtain reliable projections of extreme sea levels over the 21^st^ century. Importantly, we have utilized state of the art model data representing tide, surge, wave setup and relative sea level rise, demonstrating the contributions of each of these processes to extreme sea level and resulting episodic coastal flooding. This study builds on several previous model studies which have included some, but not all, of these elements.

Muis et al ( 8) adopted an approach similar to the present work but did not include *WS*. The resulting *TSL* was validated against a less extensive tide gauge dataset than the present study and used only a single EVA approach (AM with GUM). Based on the present results, ignoring *WS* means that $ESL_{T+S}^{H100}$ will be underestimated by between 0.3 m and 0.7 m, depending on the region under consideration. Overall, however, it is concluded that *WS* contributes on average 17% to $ESL^{H100}$ and only approximately 5% to total area flooded. The choice of EVA, however, has a significant impact on these quantities.

Vitousek et al ( 7) include *WS* and applied a single EVA approach (AM with GEV). The resulting *TSL* values were not validated either for ambient or extreme conditions and resulting coastal flooding was not investigated therein. Rather, the study concentrated on investigations of the changes in frequency of occurrence of future *ESL*.

Reuda et al ( 11) considered present day *ESL* including *WS* using a GEV distribution applied to an r-largest dataset, However, the study did not validate the data, nor did it consider projections of future *ESL*.

Vousdoukas et al ( 9) used a 6-member ensemble of models forced with Global Circulation Models to account for changes in storm frequency and magnitude in the future. These elements were combined in a Monte Carlo analysis to determine extremes. Their analysis assumed that wave setup could be represented by $WS=0.2H_{s}$and that storms always occur at high tide. No validation was undertaken to assess the performance of the model.

Melet et al ( 10) considered the contributions of tide, surge and wave setup to historical *TSL* data. No EVA was undertaken and the focus of the paper was on the importance of *WS*. As indicated by Aucan et al ( 20), this analysis assumes that global bed slope is 1/10, uses a relationship to determine *WS* which is quite sensitive to bed slope ( 18) and also includes swash (runup). Hence, the resulting contribution to *WS* is larger than may otherwise be expected. This point is discussed in more detail in SM5 below.

It should be noted that the approach used in the present analysis is to attempt to reconstruct a long duration time series of *TSL* and then perform EVA on this time series. As *T* is deterministic and *S*+*WS* is stochastic, this approach implicitly assumes that the time series is sufficiently long (36-years) to capture the random phasing of these components. That is, that extremes occur at all phases of the tide. The alternative is to consider the pdf of each process separately and combine these in a Monte-Carlo analysis ( 9).

1. **Limitations and uncertainty estimates**

Due to the global-scale focus of this work, it has been necessary to make a number of assumptions in order to render the problem computationally tractable. Obviously, these assumptions will have some impact on the accuracy of the final projections. This section outlines these assumptions and makes order-of-magnitude estimates of potential errors:

- *TSL* is considered to be the linear summation of *T+S+WS*, with each of the components evaluated independently*.* Therefore, the analysis ignores the influence tidal stage will have on *S* and *WS* ( 51, 4). It is believed these non-linear effects will be small compared to the accuracy of the EVA analysis ( 9, 52, 53, 54). Also, the extensive validation indicates that at global-scale, the present approach agrees well with tide gauge data both for ambient and extreme conditions.
- The model of *T+S+WS* is validated against tide gauges. Tide gauges which are offshore or in sheltered locations are likely to under-estimate *WS* ( 10, 55, 19). This may account for some of the scatter in the comparisons between model and tide gauge data, although the agreement is generally good.
- Model data for the period 1979-2014 is used to determine $ESL^{H100}$*.* It is then assumed that these same extreme sea levels apply in 2100. This assumes that the meteorological and ocean wave conditions do not change significantly over this period. Although there is some evidence that changes may occur ( 56, 57, 58, 59, 60, 61), determining comprehensive projections of changes in extreme values of global surge and wave height ( 62) is still an evolving research area.

Mori et al. ( 38) have estimated changes in $ESL^{F100}$for RCP8.5 using a large ensemble of Global Climate Models (GCMs). This ensemble had a spatial resolution of 60 km, subsequently downscaled to 20 km. Storm surge values were calculated in an approximate manner using an empirical relationship relating storm surge to wind speed and atmospheric pressure drop. The approach could not estimate actual values of $ESL^{F100}$but provided estimates of percentage changes in $ESL^{H100}$(i.e. changes from the present day). For mid latitudes ($\pm30^{0}$), they indicated a decrease in storm surge values of approximately 10% in the Southern Hemisphere and an increase between 10% and 30% in the Northern Hemisphere. These changes were attributed to changes in the frequency and intensity of tropical cyclones. For higher latitudes (both hemispheres) they estimated smaller increases in storm surge (approximately 10%). It should be noted that the Mori et al. ( 38) analysis did not include tide (*T*) and hence cannot be directly related to the present analysis. It should also be pointed out that there is still considerable debate about potential changes in tropical cyclone frequency, intensity and track due to climate change ( 63). Nevertheless, this analysis suggests possible impacts of up to 20% (noting the impacts of tide) on values of $ESL^{F100}$.

Morim et al ( 39) and Meucci et al ( 64) have investigated changes in global wave climate from an ensemble of GCMs which were used to force spectral wave models. They show increases in 99^th^ percentile and 100-year return period significant wave height of up to 15%, mainly in the Southern Ocean. The present analysis shows that *WS* contributes only approximately 5% to total flooding. Therefore, an increase in future extreme wave heights is unlikely to have an appreciable contribution to global flooding.

- It is assumed that $ESL^{F100}=ESL^{H100}+RSLR$*.* In a similar fashion to the *TSL*, this assumes that changes in water depth caused by the *RSLR* do not have a significant impact on extreme values of *T*, *S* or *WS* ( 65). Noting the accuracy with which the EVA can be conducted, such interactions are likely to have a secondary effect on the results for most locations ( 54, 59, 66).
- The estimates of *WS* are considered to be order of magnitude. As noted, even the GOW2 model cannot resolve bed slope or nearshore refraction and shoaling. In addition, there are a wide range of possible relationships which can be used to determine *WS*. A common engineering approach ( 16, 17) (Shore Protection Manual – SPM) has been used here. An alternative approach which has been used in other studies ( 7, 10) is that of Stockdon, et al. ( 18). In order to test the sensitivity of projected values of *WS* to the choice of this relationship, the full global analysis for the determination of $ESL^{H100}$was repeated using Stockdon, et al. ( 18) and a range of different values of bed slope. Bed slopes of 1/100, 1/30, 1/15 and 1/10 were tested, the value of 1/10 allowing a comparison with the study of Melet et al. ( 10). Fig S12a shows a comparison between $ESL^{H100}$at DIVA points using the SPM approach ( 16, 17) and Stockdon et al. ( 18), both with a bed slope of 1/30. There is very little difference in the values of *WS* between the approaches and hence the $ESL^{H100}$values all agree well*.* Fig. S11b shows the comparison between the SPM approach with a bed slope of 1/30 and Stockdon et al. with a bed slope of 1/10, as used by Melet et al. ( 10). The Stockdon et al. approach is sensitive to bed slope, increasing significantly as bed slope increases. Fig. S11b shows that with a bed slope of 1/10, the Stockdon et al. approach will predict extreme water levels , on average, 58% higher than the SPM approach with a bed slope of 1/30. This explains why Melet et al. ( 10) predict a much larger contribution due to wave setup than the present study. Note that Atanasiou et al. ( 47) indicate that the mean global bed slope is approximately 1/100 and the present analysis indicates that a value of 1/30 agrees well with tide gauge data, irrespective of which formulation is used for *WS* (see SM2). We therefore conclude that the present study uses a global mean bed slope consistent with measurements and that the choice of relationship between deep water significant wave height and *WS* makes no appreciable difference at such representative bed slopes.

It should also be noted that wave runup is not included in the present analysis. This is because wave runup does not cause an elevation in the mean sea level ( 20). In localized cases where wave runup causes overtopping it may result in localized flooding. However, such considerations are considered insignificant at global scale.

- It is assumed that no flood protection measures, such as dykes or sea walls, are in place. It is known that global flood extent is very sensitive to the assumption of current flood protection standards, but to date a comprehensive global database of such measures does not exist. Moreover, we apply a simplistic flooding model that ignores flood wave attenuation due to land roughness and an infinite flood duration (bathtub model), which will generally overestimate the flood extent ( 41, 42). So as to not mislead readers, the fact that no flood protection is considered is emphasized throughout the paper. Also, in the abstract and conclusions, changes in values between the present and 2100 are stressed rather than absolute values.
- As the MERIT ( 36) topographic model used here to derive projections of episodic coastal flooding has a spatial resolution of 1 km, small-scale features such as estuaries and rivers are not resolved. Hence, estuarine flooding will be ignored, potentially resulting in an under-estimation of the coastal flooding in some regions ( 67). The vast majority of previous coastal flooding studies ( 5, 6, 8, 24, 68) have used SRTM ( 25) topography, which has a vertical resolution of 1 m. In contrast, MERIT provides much enhanced vertical resolution. The lower resolution SRTM was also tested as part of this study. This showed large differences at specific locations, but when averaged over all global locations the differences were at most 10%.

In addition, to potential errors due to the assumptions made above, there is statistical uncertainty in the IPCC values of *RSLR* and in the extreme sea level estimates, $ESL^{H100}$. Such statistical uncertainty is usually represented in terms of confidence limits for$ESL^{H100}$(see Methods). These confidence limits provide the basis to set upper and lower bounds on the projected global land area subject to episodic coastal flooding. The global gridded *RSLR* values include 90^th^ percentile confidence limits, enabling such limits to be determined at each DIVA point. Similar confidence limits can be determined for $ESL^{H100}$as outlined in Methods. The full analysis process to determine the global area flooded was repeated for all combinations of upper, mean and lower confidence limits of both quantities. The resulting areas flooded in 2100 for both RCP4.5 and RCP8.5 are shown in Table S4. The results in Table S4 show that, on average, the uncertainty span (i.e. upper limit – lower limit) on the areas flooded is approximately 31.1% for *RSLR* and 11.4% for *ESL* for RCP8.5. Following Wahl et al. ( 31), these uncertainties have been combined using the sum of the squares of the individual uncertainties $\Delta_{Tot}=\sqrt{\sum_{i=1}^{N} \Delta_{i}^{2}}$, where $\Delta_{Tot}$is the total percentage uncertainty and $\Delta_{i}$is the percentage uncertainty from process $i$ (in this case either *RSLR* or *ESL*). When the above values are combined in this manner the resulting uncertainty span for flooded area is 33.1% or ±16.5%.

**References**

| 51. | Arns, A. *et al.*, Sea-level rise induced amplification of coastal protection design heights. *Sci. Rep.* **7**, 40171 (2017). |
| --- | --- |
| 52. | Vousdoukas, M. I., Mentaschi, L., Voukouvalas, E., Verlaan, M. & Feyen, L., Extreme sea levels on the rise along Europe's coasts. *Earth's Future* **5** (3), 304–323 (2017). |
| 53. | Howard, T., Lowe, J. & Horsburgh, K., Interpreting Century-Scale Changes in Southern North Sea Storm Surge Climate Derived from Coupled Model Simulations. *Journal of Climate* **23**, 6234-6247 (2010). |
| 54. | Sterl, A. *et al.*, An ensemble study of extreme storm surge related water levels in the North Sea in a changing climate. *Ocean Science* **5** (3), 369-378 (2009). |
| 55. | Hoeke, R. K., McInnes, K. L. & O’Grady, J. G., Wind and Wave Setup Contributions to Extreme Sea Levels at a Tropical High Island: A Stochastic Cyclone Simulation Study for Apia, Samoa. *Journal of Marine Science and Engineering* **3**, 1117-1135 (2015). |
| 56. | Debernard, J. B. & Roed, L. P., Future wind, wave and storm surge climate in the Northern Seas: a revisit. *Tellus* **60** (3), 427-438 (2008). |
| 57. | Wang, S. *et al.*, The impact of climate change on storm surges over Irish waters. *Ocean Modelling* **25** (1-2), 83-94 (2008). |
| 58. | Hemer, M. A., Fan, Y., Mori, N., Semedo, A. & Wang, X. L., Projected changes in wave climate from a multi-model ensemble. *Nature Climate Change* **3**, 471-476 (2013). |
| 59. | Lowe, J. A., Gregory, J. M. & Flather, R. A., Changes in the occurrence of storm surges around the United Kingdom under a future climate scenario using a dynamic storm surge model driven by the Hadley Centre climate models. *Climate Dynamics* **18** (3-4), 179-188 (2001). |
| 60. | Young, I. R., Zieger, S. & Babanin, A. V., Global trends in wind speed and wave height. *Science* **332**, 451-455 (2011). |
| 61. | Young, I. R. & Ribal, A., Multi-platform evaluation of global trends in wind speed and wave height. *Science* (eaav9527) (2019). |
| 62. | Mentaschi, L., Vousdoukas, M. I., Voukouvalas, E., Dosio, A. & Feyen, L., Global changes of extreme coastal wave energy fluxes triggered by intensified teleconnection patterns. *Geophys. Res. Lett.* **44**, 2416–2426 (2017). |
| 63. | Patricola, C. M. & Wehner, M. F., Anthropogenic influences on major tropical cyclone events. *Nature* **563**, 339-346 (2018). |
| 64. | Meucci, A., Young, I. R., Hemer, M. . K. E. & Ranasinghe, R., Global extreme ocean wave heights magnitude and frequency projected to the end of the 21 Century. *Science Advances*, **6**, eaaz7295 (2020). |
| 65. | Pickering, M. D. *et al.*, The impact of future sea-level rise on the global tides. *Cont. Shelf. Res.* **142**, 50-68 (2017). |
| 66. | McInnes, K. L., Macadam, I., Hubbert, G. & O'Grady, J., An assessment of current and future vulnerability to coastal inundation due to sea‐level extremes in Victoria, southeast Australia. *International Journal of Climatology* **33** (1), 33-47 (2013). |
| 67. | Kulp, S. & Strauss, B. H., Global DEM errors underpredict coastal vulnerability to sea level rise and flooding. *Front. Earth Sci.* **4**, 36 (2016). |
| 68. | Neumann, B., Vafeidis, A. T., Zimmermann, J. & Nicholls, R. J., Future Coastal Population Growth and Exposure to Sea-Level Rise and Coastal Flooding - A Global Assessment. *PLoS ONE* **10:e0118571** (2015). |

**Fig. S1.** Global plot of DIVA output locations (purple points) and GESLA-2 tide gauge locations (yellow points). (Figure generated using ArcGIS v.10.5.1.7333, www.esri.com).


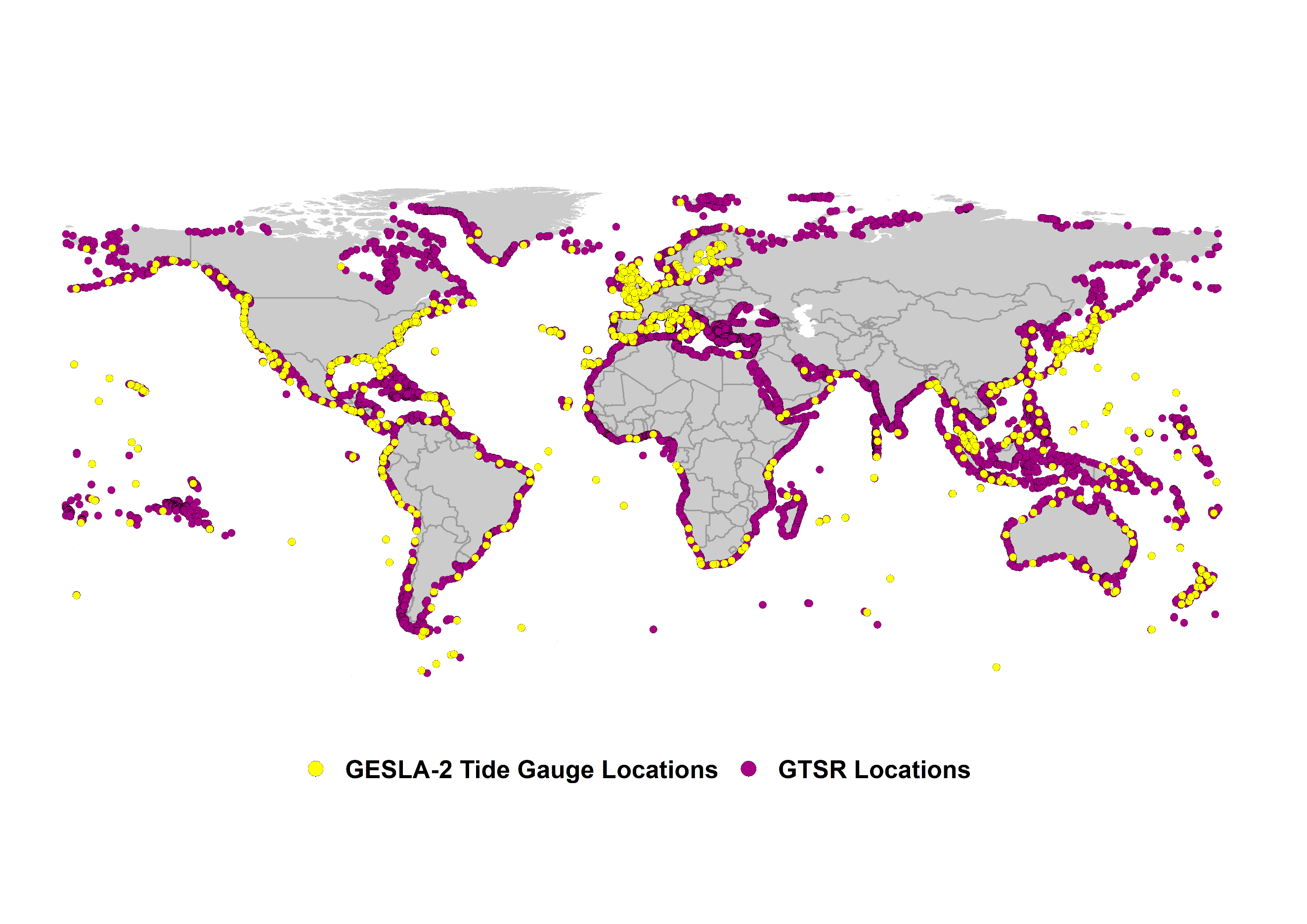


**Fig. S2.** Values of root mean squared error (*RMSE*) between model total sea level (*TSL=T+S+WS*) and GESLA-2 tide gauge data. (Figure generated using ArcGIS v.10.5.1.7333, www.esri.com).


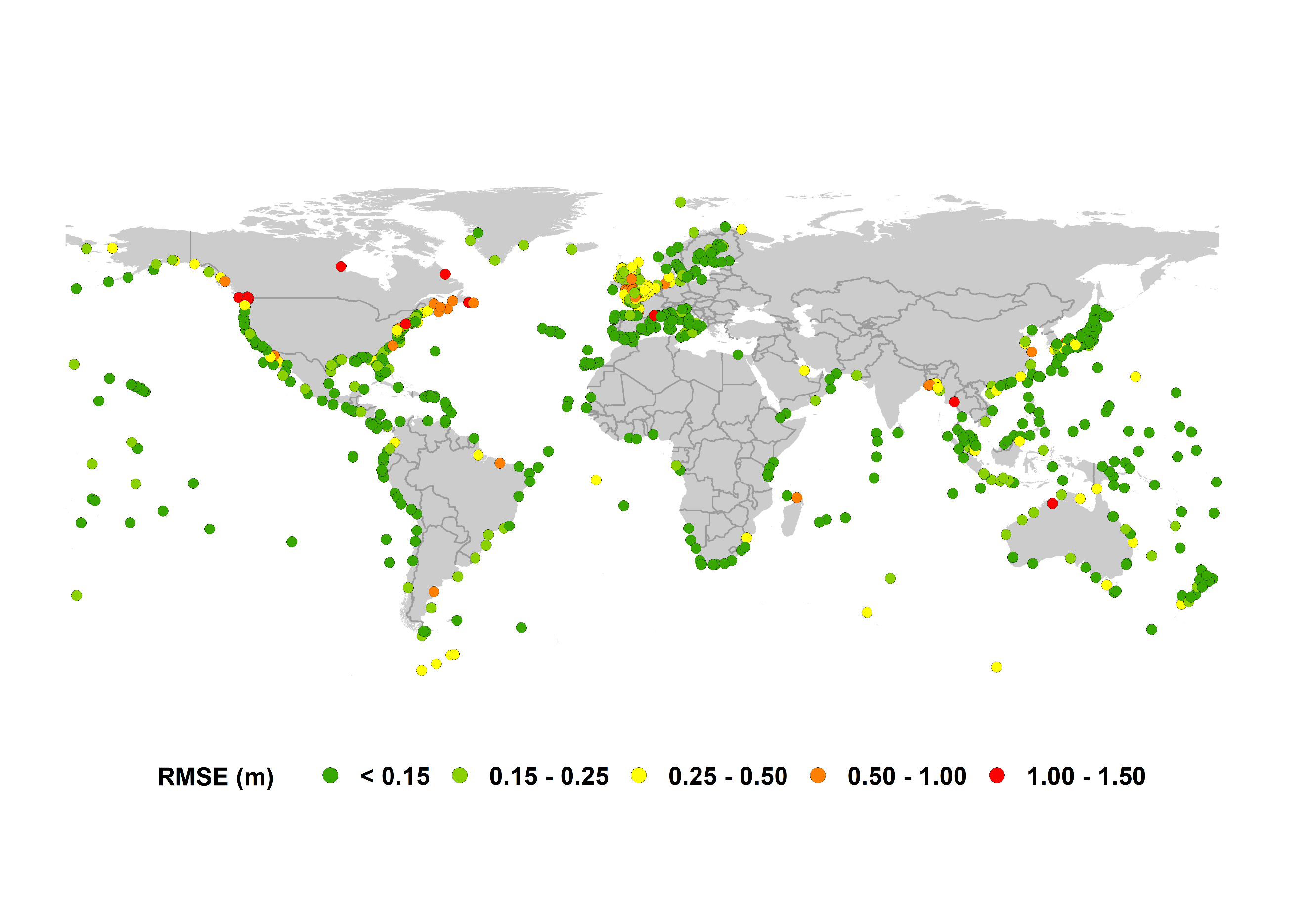


**Fig. S3.** Values of root mean squared error (*RMSE*) between model total sea level (*TSL=T+S+WS*) and GESLA-2 tide gauge data (as in Fig. S2) as a percentage of the tidal amplitude at that location. (Figure generated using ArcGIS v.10.5.1.7333, www.esri.com).


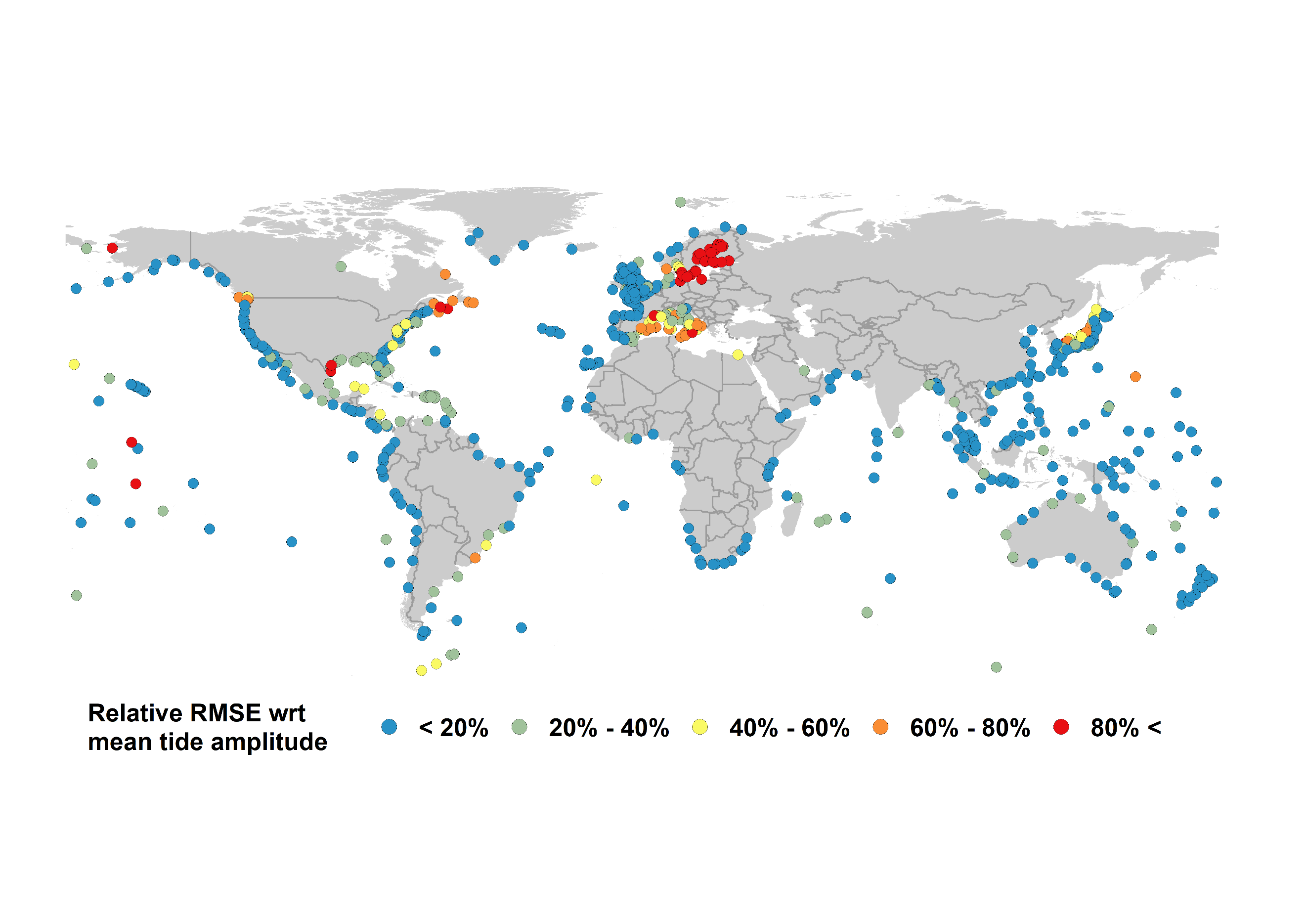


**Fig. S4.** Change in $\left|bias^{P}\right|$ at the 99^th^ percentile between model and tide gauge as a result of wave setup (*WS*). i.e., $\left|bias_{T+S+WS}^{P}\right|-\left|bias_{T+S}^{P}\right|$ evaluated at GESLA-2 tide gauge locations. Negative (blue) values indicate an improvement in agreement between model and tide gauge for extreme values as a result of the inclusion of *WS.* (Figure generated using ArcGIS v.10.5.1.7333, www.esri.com).


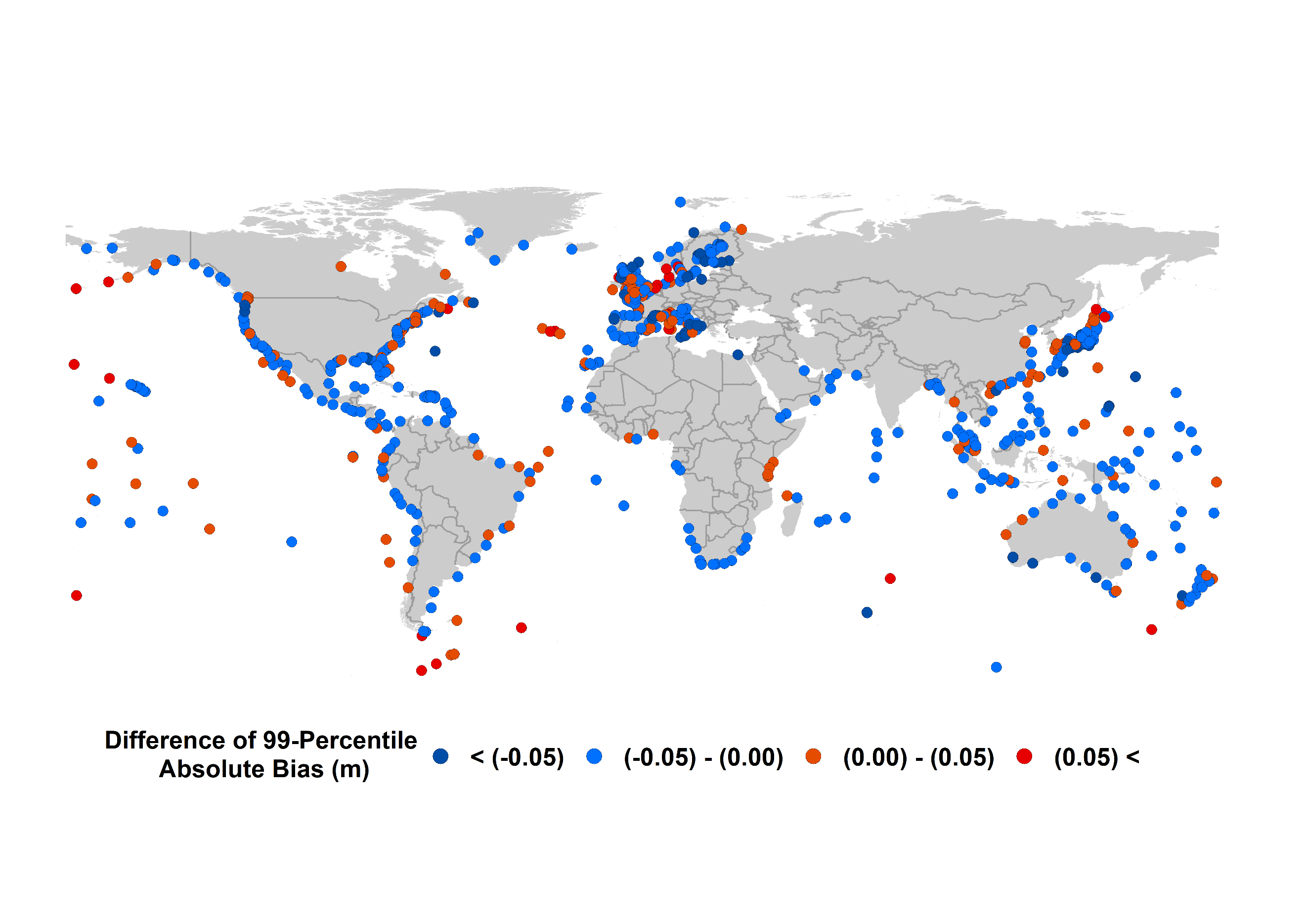


**Fig. S5.** Best fitting EVA approach at GESLA-2 tide gauge locations. The color of the marker at each location indicates the EVA approach which best fits the tide gauge pdf at that location. The colour bar legend defines each of the EVA approaches (e.g. GPD P98 is the Generalized Pareto Distribution with a 98^th^ percentile). The histogram (top left) shows the relative number of points where each EVA approach gives the best fit to the tide gauge pdf. Only locations with more than 20-years of data shown. (Figure generated using Matlab 2018a, https://www.mathworks.com/products/matlab/).


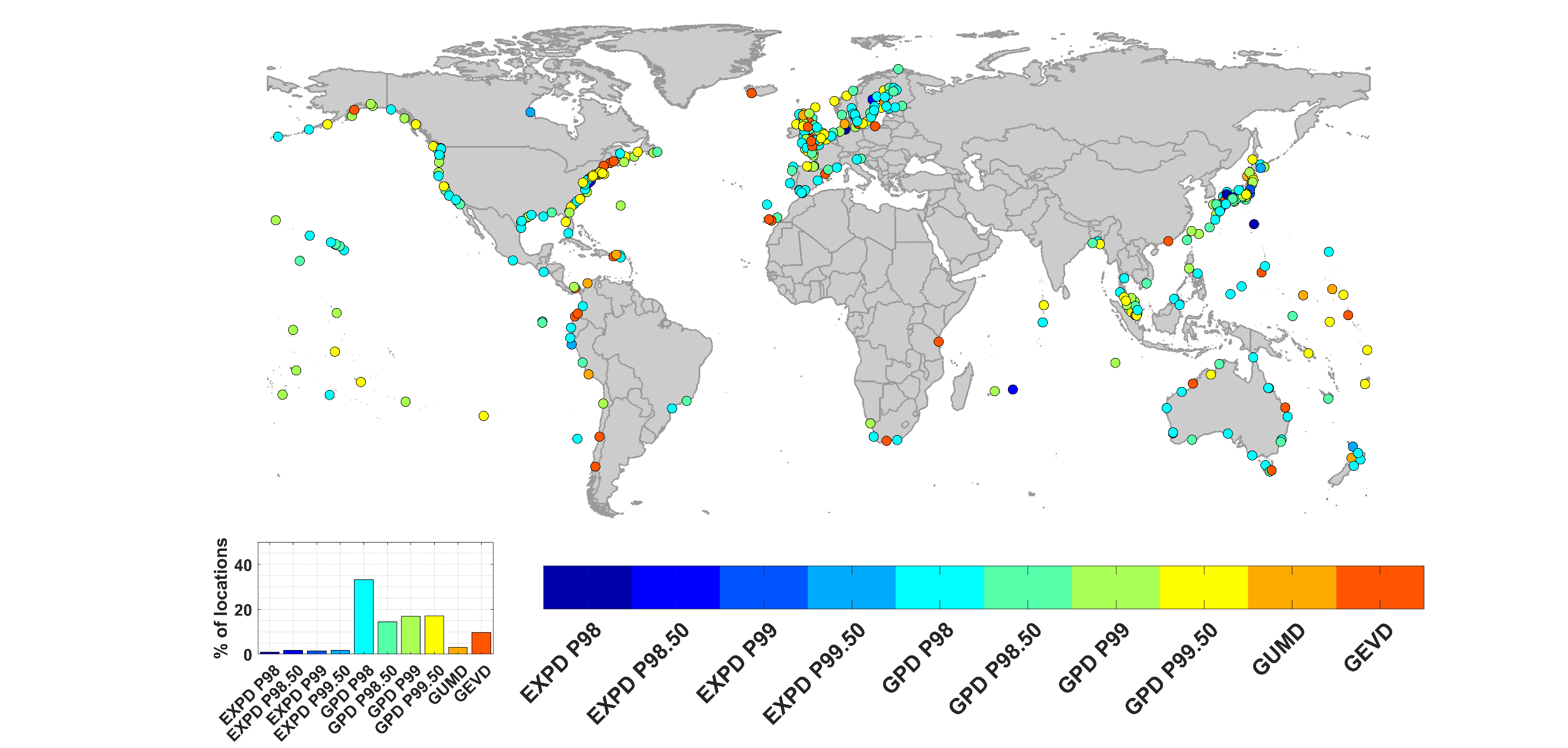


**Fig. S6.** Global distribution of the contribution of wave setup to the 100-year return period extreme sea level (i.e. $ESL_{T+S+WS}^{H100}-ESL_{T+S}^{H100}$) at DIVA locations. Based on model data for the period 1979-2014. (Figure generated using ArcGIS v.10.5.1.7333, www.esri.com).


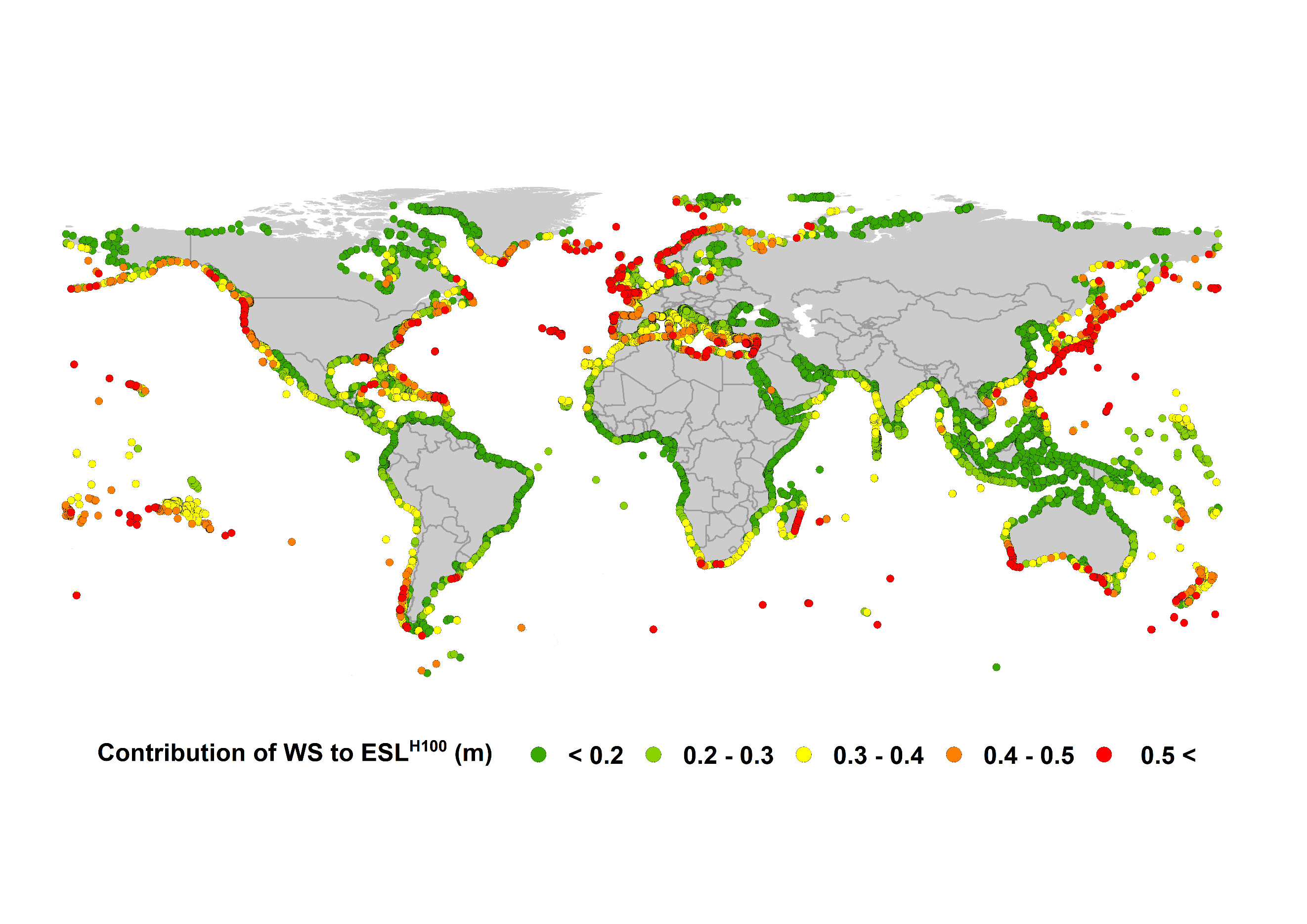


**Fig. S7.** (left) Histogram of bias for $ESL^{H20}$between GESLA-2 tide gauge data and model ($ESL_{T+S+WS}^{H20}-ESL_{Gauge}^{H20}$) (blue bars) and ($ESL_{T+S}^{H20}-ESL_{Gauge}^{H20}$) (unfilled bars). (right) The bias of the 99^th^ percentile values of *TSL* between model and GESLA-2 tide gauge data, $bias^{p}$. *T+S+WS* shown by blue bars, *T+S* shown by unfilled bars. The inclusion of *WS* reduces the absolute magnitude of both the $ESL^{H20}$average bias and the average 99^th^ percentile $bias^{p}$ (i.e. more centred around a value of zero).


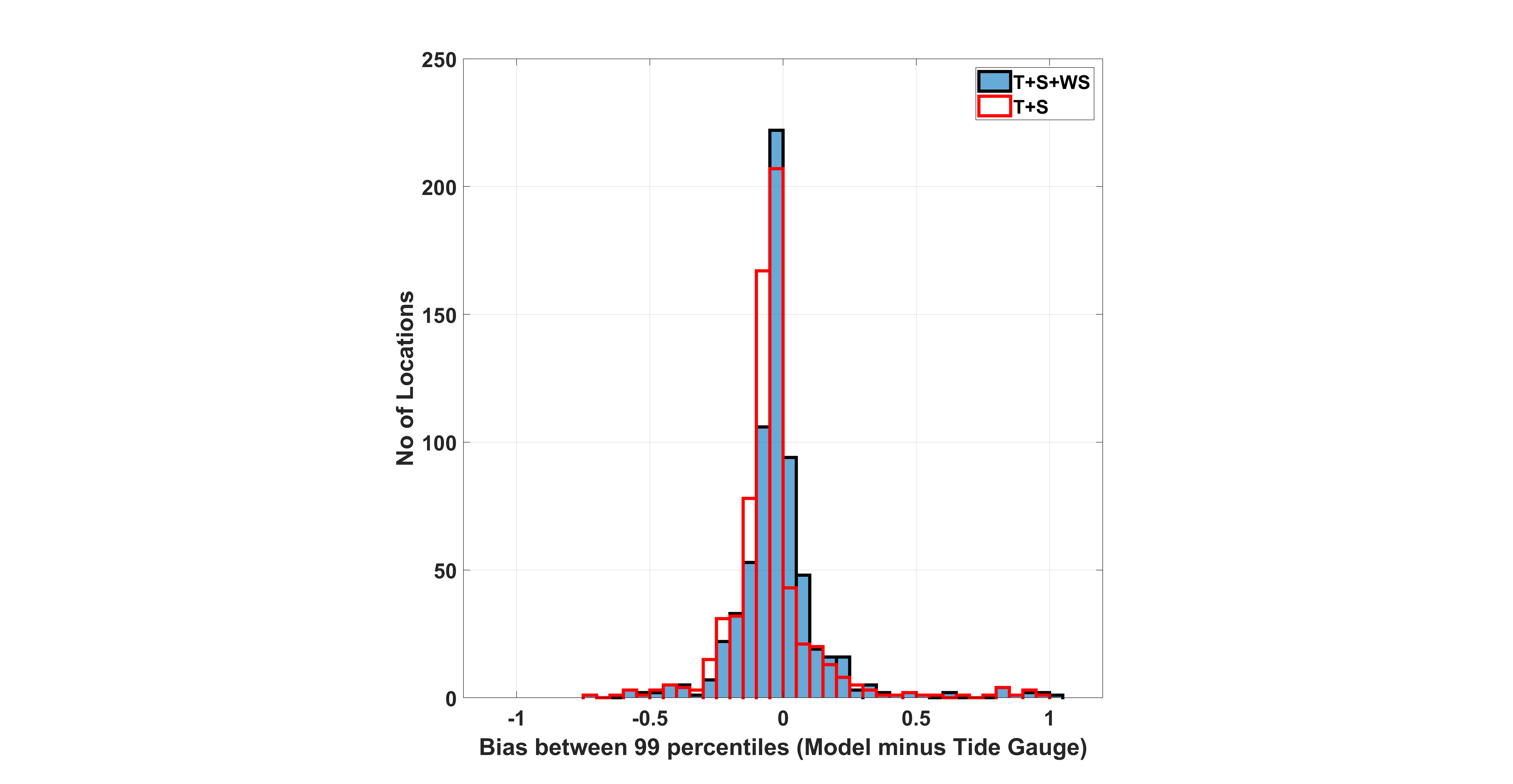


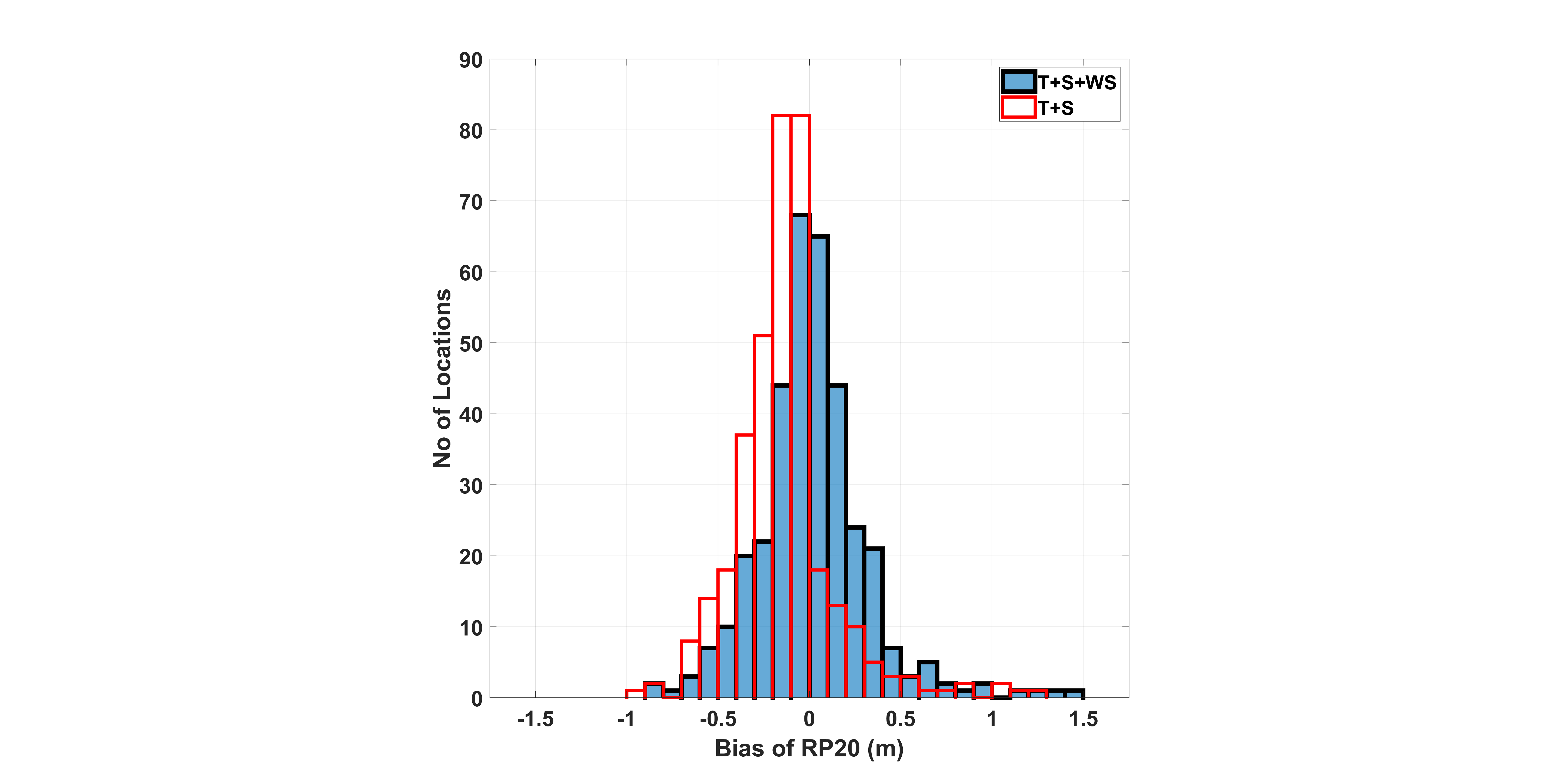


**(a)**

**Fig. S8.** Residual sea level and significant wave height as a function of time at Boston, USA. (a) Values over the year 2013. (b) Details of the storm event during February 2013. (top) Model storm surge (S) (blue line) and model surge + wave setup (*S+WS*) (red line). (bottom) GOW2 significant wave height during the same storm event.


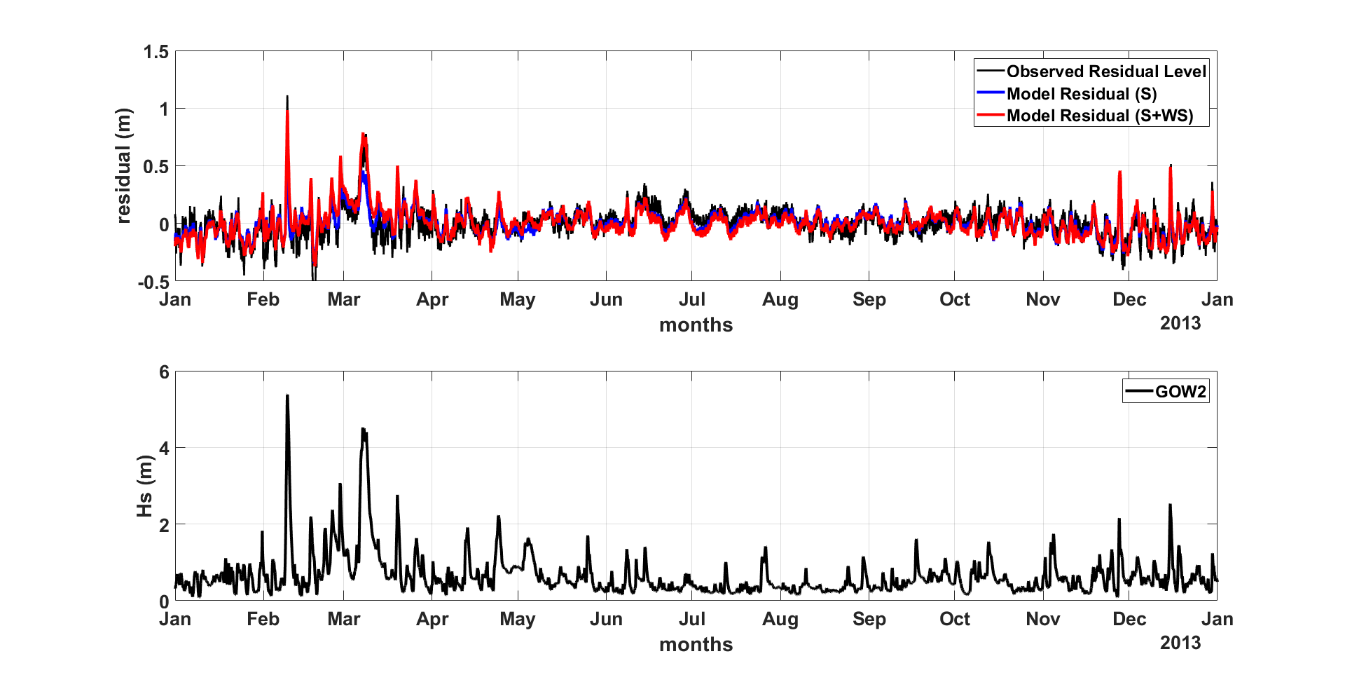


**(b)**


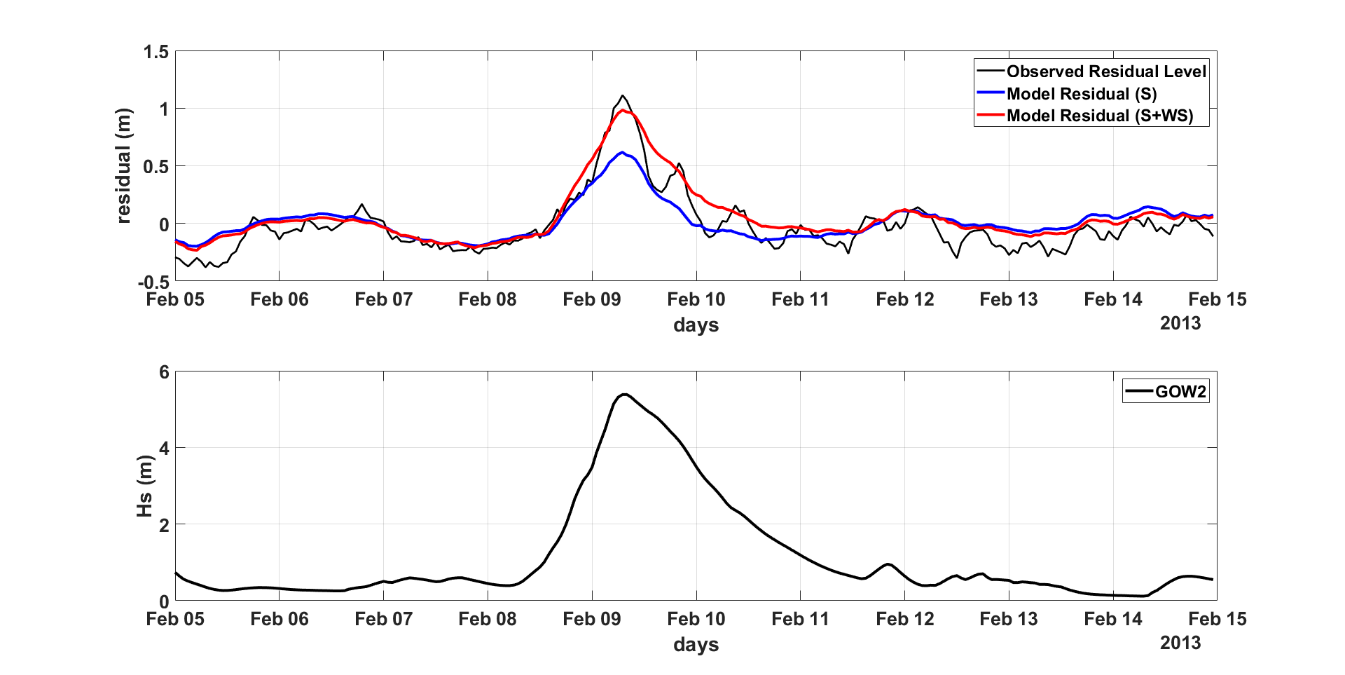


**(b)**

**Fig. S9.** Residual sea level and significant wave height as a function of time at Fremantle, Australia. (a) Values over the year 2009. (b) Details of the storm event during June 2009. (top) Model storm surge (S) (blue line) and model surge + wave setup (*S+WS*) (red line). (bottom) GOW2 significant wave height during the same storm event.


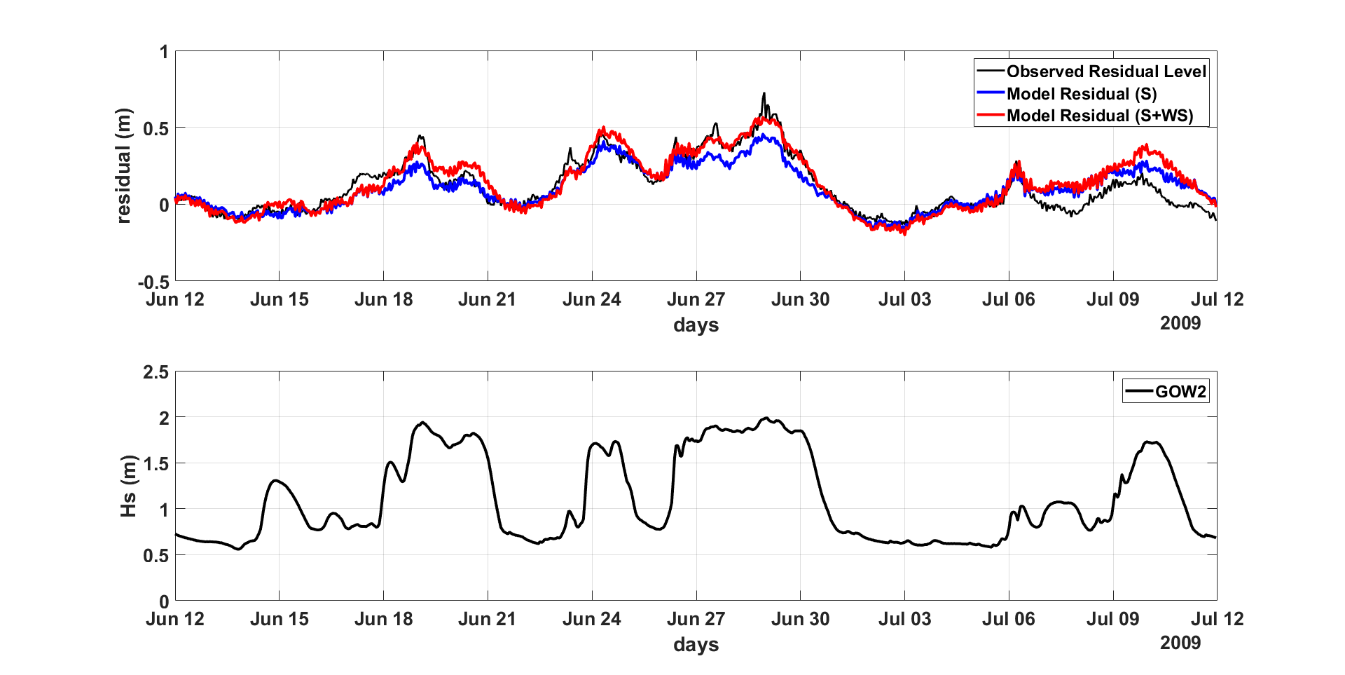


**(a)**


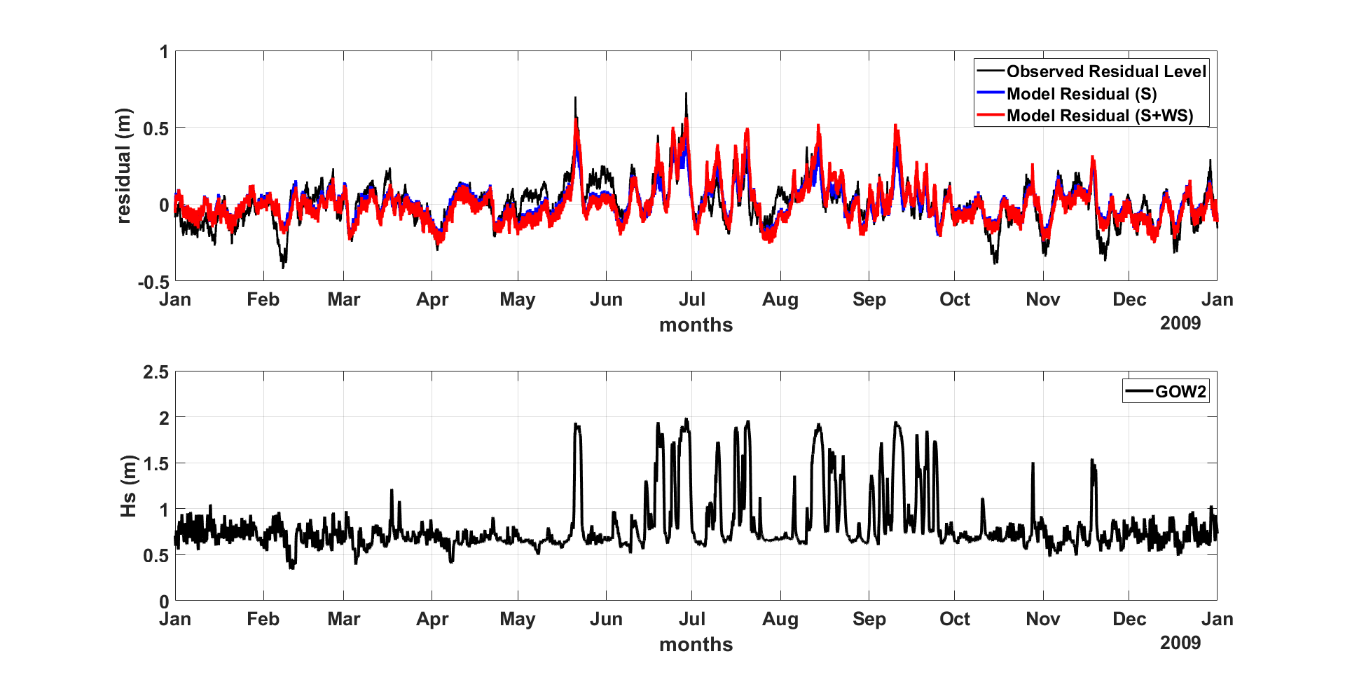


**Fig. S10.** Extreme value total sea level at Boston, USA as a function of return period. Exponential distribution (EXP) shown on the left and Generalized Pareto Distribution (GPD) on the right. Tide gauge data are shown with the dashed lines. Model results with no wave setup shown with triangles and with wave setup included as filled circles. Different threshold percentile levels for EVA shown with colours defined in the colour bar.


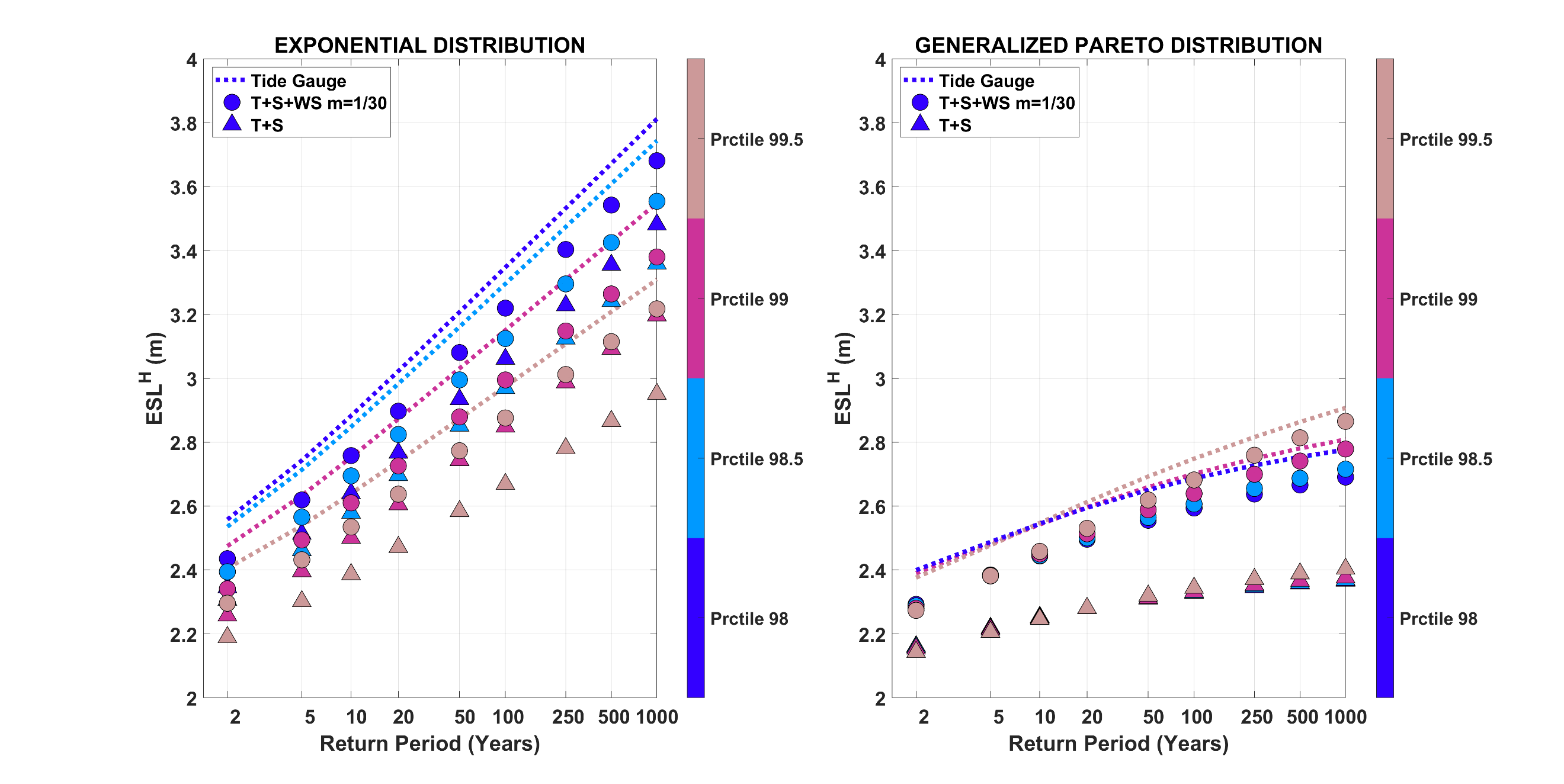


**(b)**

**(a)**

**Fig. S11.** Comparison between Extreme Sea Levels ($ESL^{H100}$) determined with different formulations for *WS*. (left) Comparison between SPM ( 16, 17) and Stockdon et al. ( 18) with a bed slope of 1/30 for both. (right) Comparison between SPM ( 16, 17) with bed slope of 1/30 and Stockdon et al. ( 18) with a bed slope of 1/10 (as used by Melet et al. ( 10).

**Fig. S12.** Global distribution of projected extreme sea level ($ESL_{T+S+WS}^{F100}$) for RCP8.5 in (A) 2050 and (B) 2100. (Figure generated using ArcGIS v.10.5.1.7333, www.esri.com).


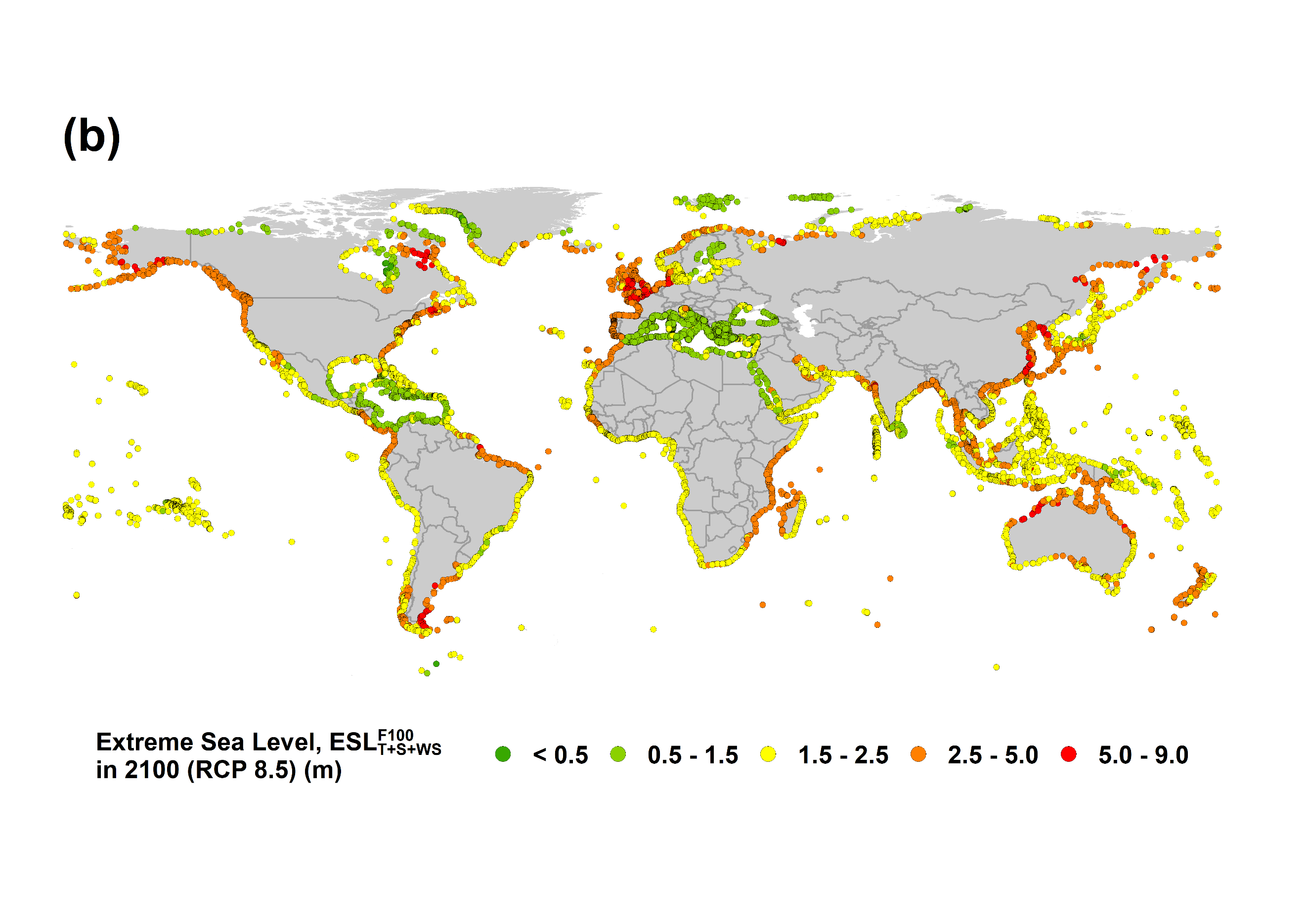


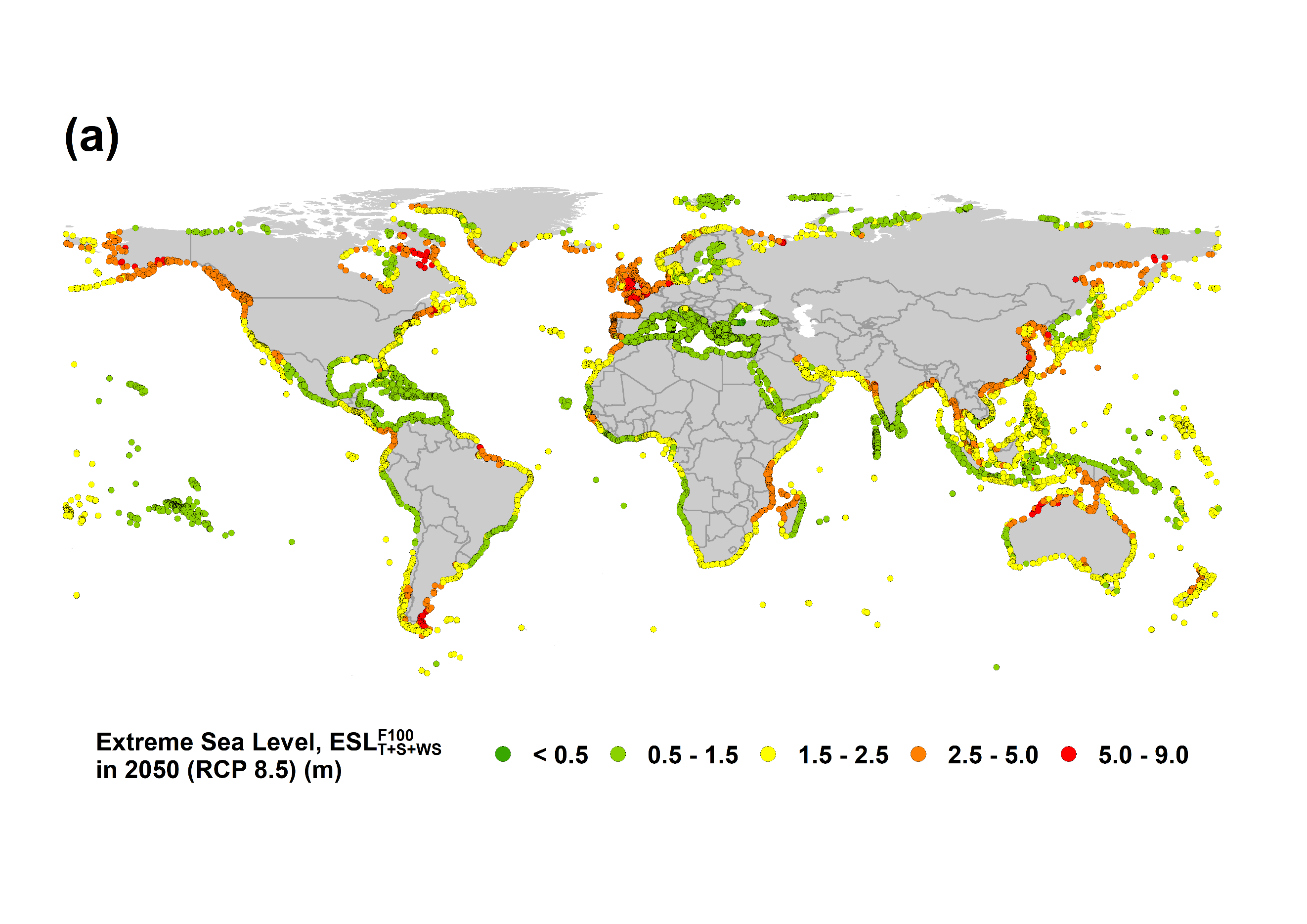


| ***ARMSE* and *abias^P^* between Model and Tide Gauge *TSL* (m)** | | | | | | | |
| --- | --- | --- | --- | --- | --- | --- | --- |
|  | **No WS** | **ERAI-15** | **ERAI-30** | **ERAI-100** | **GOW2-15** | **GOW2-30** | **GOW2-100** |
| ***ARMSE*** | 0.197 | 0.205 | 0.204 | 0.203 | 0.205 | 0.204 | 0.203 |
| ***abias^P^*** | -0.044 | -0.010 | -0.014 | -0.017 | -0.013 | -0.017 | -0.019 |

**Table S1.** Average root mean squared error (*ARMSE*) and average bias of 99^th^ percentile values (*abias^P^*) for *TSL* between model and GESLA-2 tide gauge data. Model values are shown with and without *WS* calculated using either GOW2 or ERA-I wave models and a variety of bed slopes. [e.g. GOW2-30 indicates GOW2 wave model and bed slope of 1/30 – other combinations similarly named).

|  | ***abias^P^* between the percentiles of the Model and Tide Gauge *TSL* (m)** | | | | |
| --- | --- | --- | --- | --- | --- |
|  | **95^th^** | **96^th^** | **97^th^** | **98^th^** | **99^th^** |
| ***'T+S'*** | -0.031 | -0.033 | -0.035 | -0.039 | -0.044 |
| ***'T+S+WS'*** | -0.019 | -0.019 | -0.019 | -0.019 | -0.017 |

**Table S2.** Average bias (*abias^P^*) of percentile values for *TSL* between model and GESLA-2 tide gauge data. Values for each percentile are shown for model values consisting of “*T+S*” (i.e. no *WS*) and “*T+S*+*WS*”. Negative values indicate that model is less than tide gauge. Note that to calculate *WS*, the GOW2 model was used, and a bed slope of 1/30.

|  | **GUM** | **GEV** | **GPD98** | **GPD98.5** | **GPD99** | **GPD99.5** | **EXP98** | **EXP98.5** | **EXP99** | **EXP99.5** |
| --- | --- | --- | --- | --- | --- | --- | --- | --- | --- | --- |
| **No WS** | -0.141 | -0.153 | -0.136 | -0.138 | -0.139 | -0.144 | -0.081 | -0.077 | -0.080 | -0.097 |
| **ERAI-15** | 0.046 | 0.037 | 0.036 | 0.043 | 0.051 | 0.061 | 0.040 | 0.033 | 0.023 | 0.023 |
| **ERAI-30** | 0.024 | 0.017 | 0.015 | 0.021 | 0.029 | 0.040 | 0.025 | 0.018 | 0.006 | 0.004 |
| **ERAI-100** | 0.011 | 0.004 | 0.002 | 0.007 | 0.015 | 0.026 | 0.016 | 0.008 | -0.004 | -0.008 |
| **GOW2-15** | 0.036 | 0.031 | 0.028 | 0.034 | 0.043 | 0.050 | 0.026 | 0.017 | 0.007 | 0.008 |
| **GOW2-30** | 0.016 | 0.011 | 0.017 | 0.013 | 0.021 | 0.030 | 0.013 | 0.004 | -0.008 | -0.009 |
| **GOW2-100** | 0.004 | -0.002 | -0.004 | 0.001 | 0.009 | 0.018 | 0.006 | -0.004 | -0.016 | -0.019 |

**Table S3.** The global mean bias between model and tide gauges for extreme sea levels ($ESL^{H20}-ESL_{Gauge}^{H20}$) using all 10 EVA approaches. Results are shown for no *WS* and with *WS* calculated either with GOW2 or ERA-I wave models and a variety of bed slopes. [e.g. GOW2-30 indicates GOW2 wave model and bed slope of 1/30; GPD98 indicates a Generalized Pareto Distribution with a 98^th^ percentile threshold].

| 1. **RCP4.5** | ***ESL_lower_* (10^3^ km^2^)** | ***ESL_mean_* (10^3^ km^2^)** | ***ESL_upper_* (10^3^ km^2^)** | **Uncertainty span** |
| --- | --- | --- | --- | --- |
| ***RSLR_lower_* (10^3^ km^2^)** | 604 | 647 | 697 | -6.5% to 7.8% |
| ***RSLR_mean_* (10^3^ km^2^)** | 699 | 737 | 789 | -5.2% to 7.0% |
| ***RSLR_upper_* (10^3^ km^2^)** | 797 | 837 | 894 | -4.8% to 6.7% |
| **Uncertainty span** | -13.5% to 14.0% | -12.3% to 13.6% | -11.6% to 13.3% |  |

| 1. **RCP8.5** | ***ESL_lower_* (10^3^ km^2^)** | ***ESL_mean_* (10^3^ km^2^)** | ***ESL_upper_* (10^3^ km^2^)** | **Uncertainty span** |
| --- | --- | --- | --- | --- |
| ***RSLR_lower_* (10^3^ km^2^)** | 661 | 700 | 750 | -5.5% to 7.2% |
| ***RSLR_mean_* (10^3^ km^2^)** | 779 | 819 | 874 | -4.9% to 6.8% |
| ***RSLR_upper_* (10^3^ km^2^)** | 915 | 956 | 1,009 | -4.3% to 5.6% |
| **Uncertainty span** | -15.1% to 17.5% | -14.5% to 16.8% | -14.2% to 15.4% |  |

**Table S4:** Uncertainty analysis for projected area flooded in 2100 accounting for statistical uncertainty in estimates of Extreme Sea Level (*ESL*) and Relative Sea Level Rise (*RSLR*). Area flooded in units of [10^3^ km^2^] is shown for the analysis using “upper”, “mean” and “lower” bound estimates for both *ESL* and *RSLR*. The “Uncertainty span” shows the percentage difference for the lower to the upper bound estimates of area flooded relative to the mean [e.g. $\left(ESL_{upper}-ESL_{mean}\right)/ESL_{mean}$ and $\left(ESL_{lower}-ESL_{mean}\right)/ESL_{mean}$]. Table (a) shows results for RCP4.5 and Table (b) for RCP8.5.
